# Supplementary material for: Spatial transcriptomics reveal impaired metabolic liver zonation in acute-on-chronic liver failure
Source: JHEP Rep. 2026 Sep 10;8(12):102035. doi: 10.1016/j.jhepr.2026.102035 (PMC13756263; doi:10.1016/j.jhepr.2026.102035)
Supplement: Supplementary file 1 [file mmc1.pdf]

Spatial transcriptomics reveal impaired metabolic liver zonation in acute-on-chronic liver failure

Mona-May Langer, Helena Stadler, Mareike Mannewitz, Alina Bauschen, Severin Jacobi, Dionysios Koliogiannis, Markus Guba, Devon Siemes, Daniel R. Engel, Carolin Mogler, Johannes Wirth, Katja Steiger, Bettina Budeus, Christian M. Lange

Table of contents

Fig. S1……………………………………………………………………………………2

Fig. S2……………………………………………………………………………………3

Fig. S3……………………………………………………………………………………4

Fig. S4……………………………………………………………………………………5

Table S1……………………………………………………..…………separate excel file

Table S2…………………………………………………………………………………6

Table S3…………………………………………………………………………………7

Table S4…………………………………………………………………………………8

Table S5…………………………………………………………………………………8

Fig. S1: Heatmap showing unsupervised clustering of the top differentially regulated genes (DRGs) across stages of cirrhosis (padj < 0.03 & abs(log2FC) > 0.65).

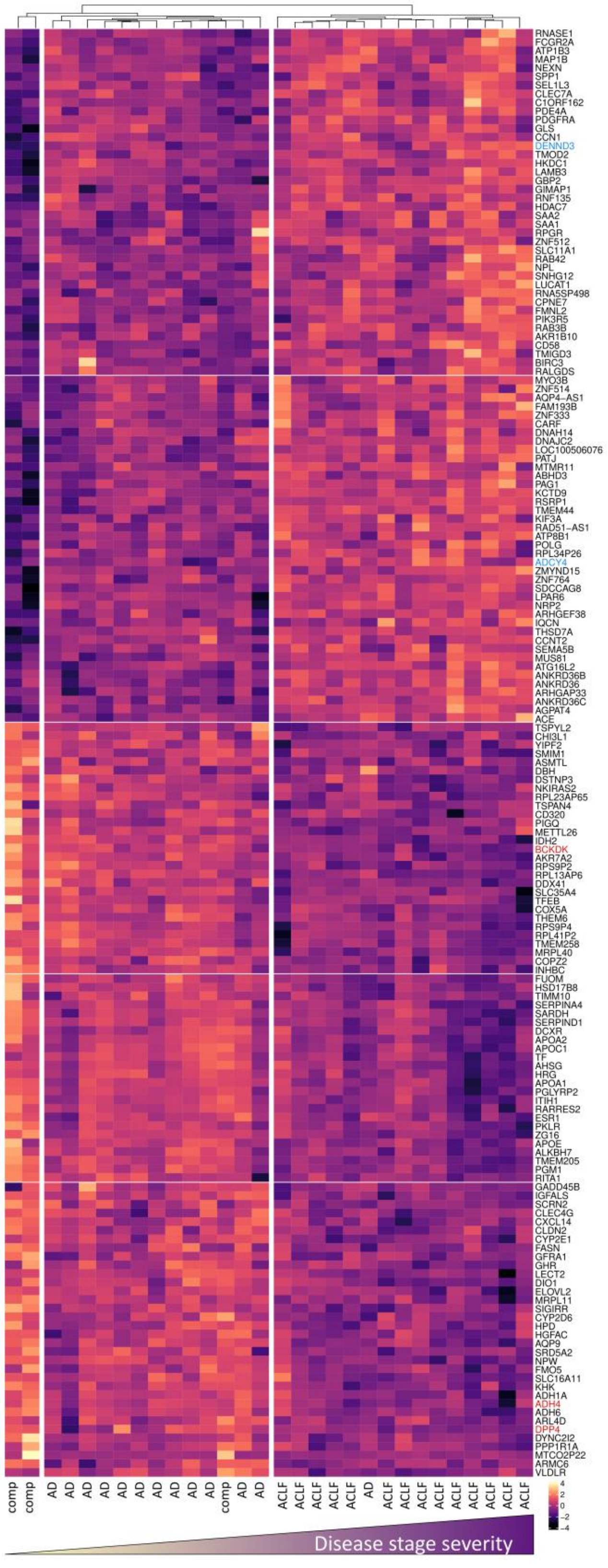

Fig. S2: Correlation of hepatic expression of selected genes with markers of systemic inflammation.

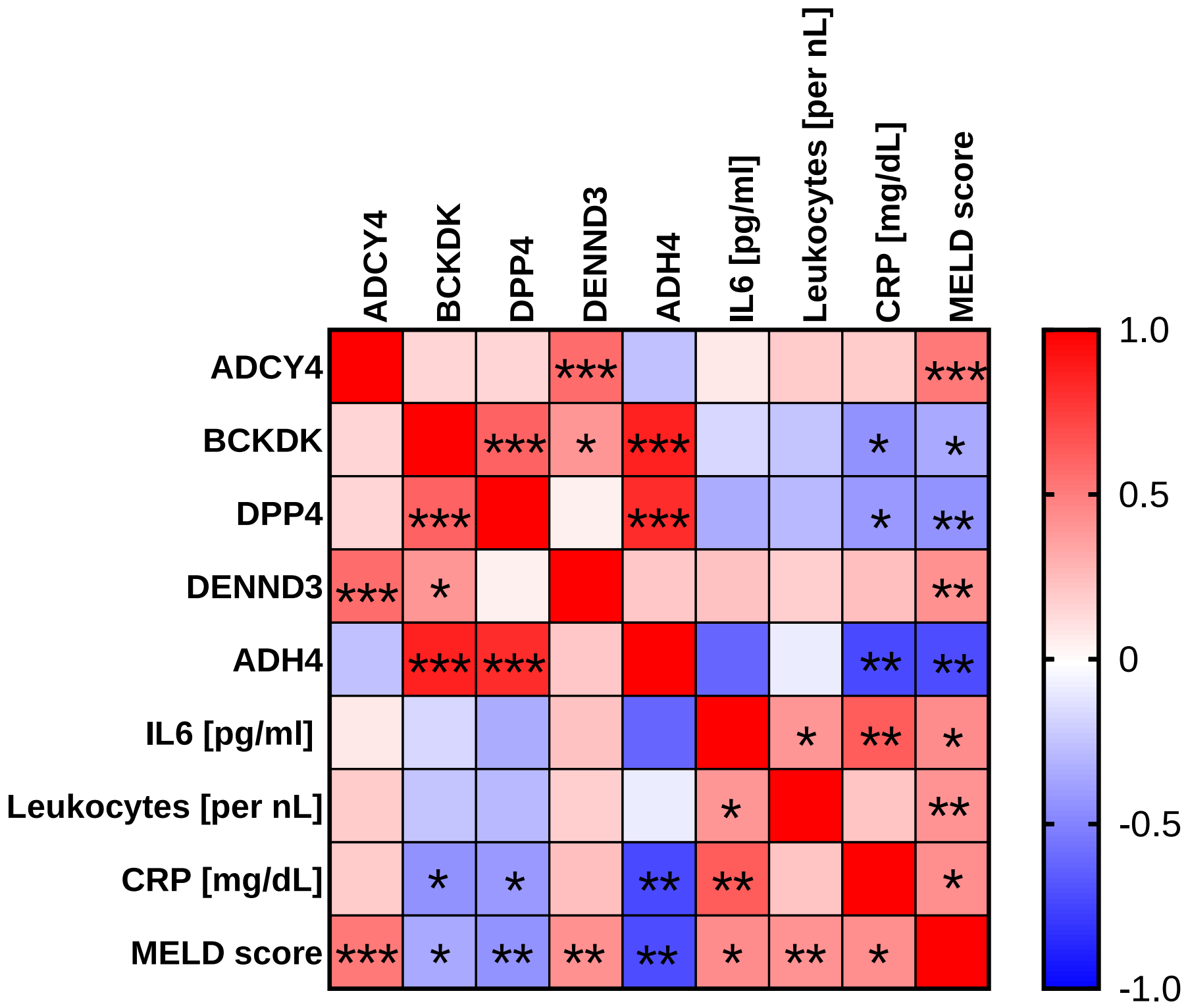

Correlations of hepatic gene expression, assessed by quantitative PCR (see Figure 1) with markers of systemic inflammation. *P ≤ 0.05, **P ≤ 0.01, ***P ≤ 0.001.

Fig. S3: Association between hepatic expression of selected genes according to the presence of infections as precipitants of ACLF.

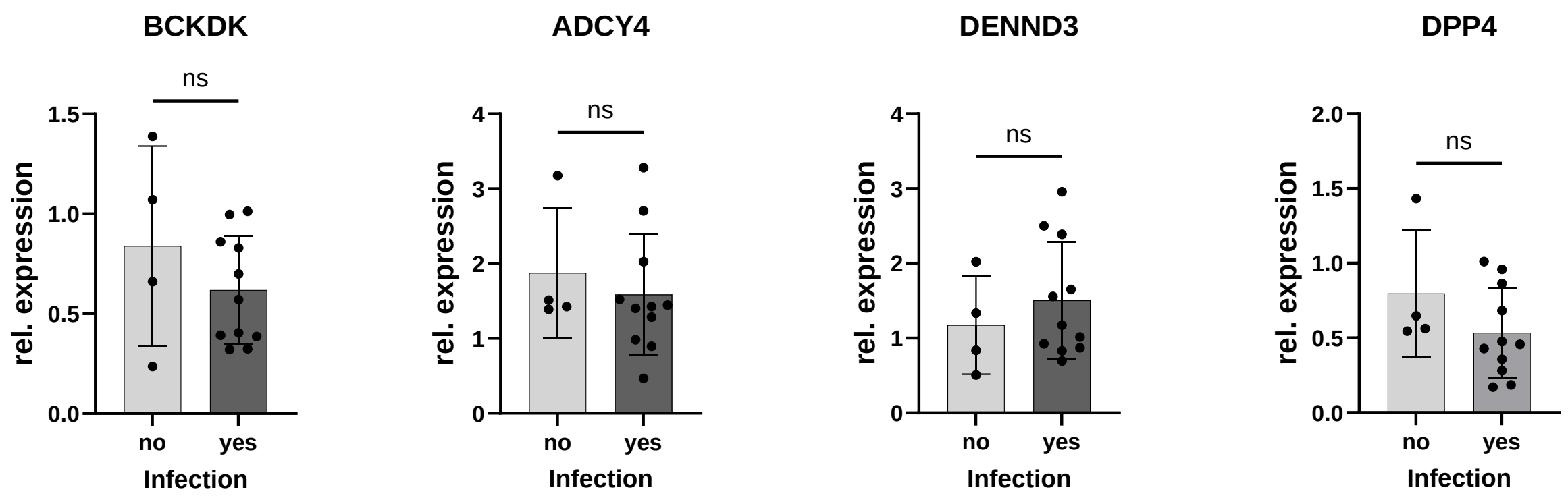

Data are shown for patients with ACLF with versus without infections.

Fig. S4: Association between hepatic expression of selected genes according to the type of organ failures.

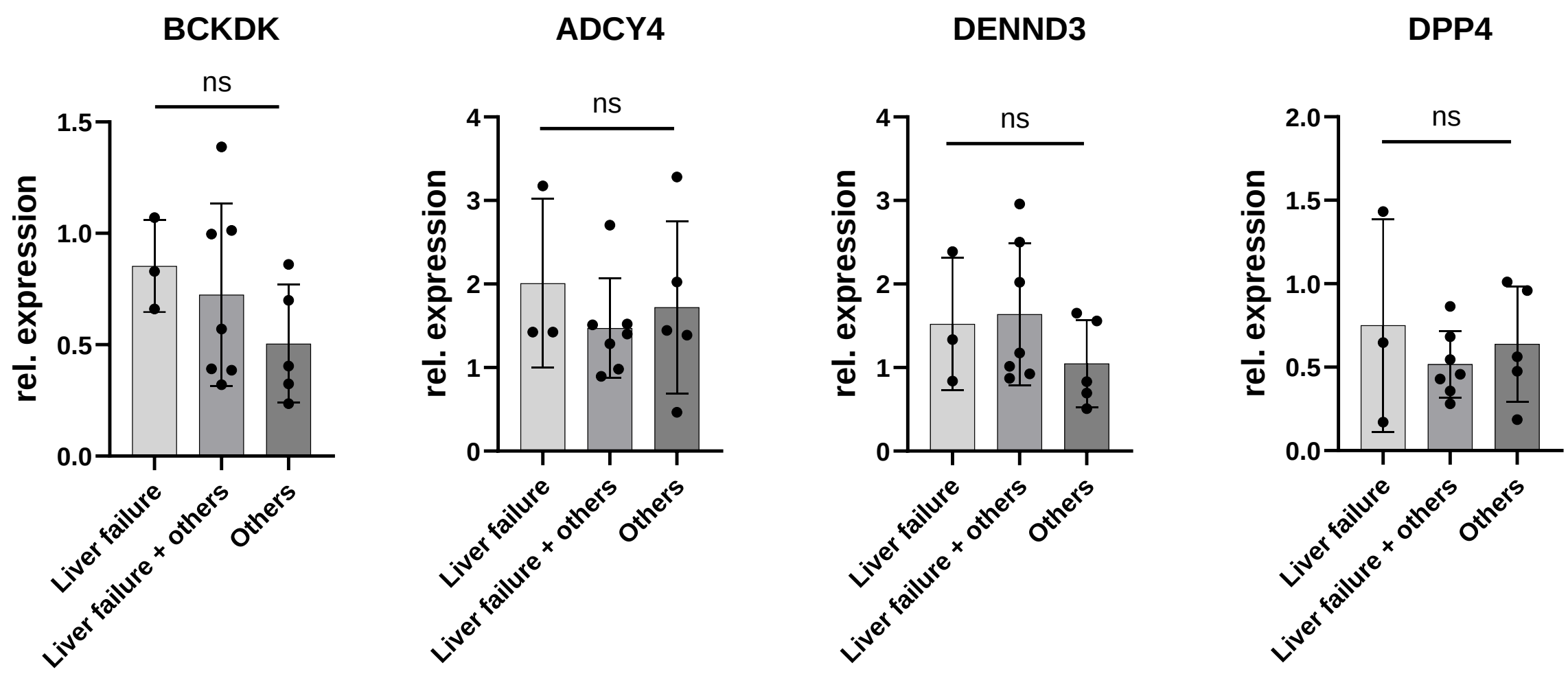

Data are shown for patients with ACLF with versus without indicated organ failures.

Table S1. List of genes included in Xenium Panel: See attached Excel sheet.

Table S2. List of genes (according to (21)) used for cell type annotation of spatial transcriptome analysis.

**Cell Type**	**Marker Genes**
Hepatocytes_drugmetabolism	ADH4, CYP3A4, RPP25L, HAMP, BCHE, G6PC
Hepatocytes_gluconeogenesis	ADH4, CYP3A4, SCD, HMGCS1
Hepatocytes_immuneactivation	ADH4, CYP3A4, SEC16B, CYP2A7
LSECs_Zone1	RAMP2, CLEC14A, VWF, C7, SRPX, CAV1, SOX18
LSECs_Zone2-3	FCN2, CRHBP, GNG11, DNASE1L3, ADGRL4, EGFL7, LYVE1
non-LSEC_endothelial_cells	PECAM1, INMT, DNASE1L3, PTGDS, C7, VWF, SRPX
Cholangiocytes	EPCAM, TFF2, FXYD2, KRT7, CXCL6
HSC	ACTA2, PDGFRB, LTBP2, HGF
Kupffer_cells	CD163, MARCO, MRC1, FCN1
B_cells	CD19, CD27, MS4A1, TCL1A, BANK1, IRF8, CD79A
Dendritic_cells	CD14, CD19, CD80, CD83, CD86, IRF8, LILRA4
Macrophages	IL1B, IL6, CD80, CD86, CD4, CD83
T_cells	CD2, CD3D, TRAC, GZMK, CCL5, GZMA, CD3E, CD69, IL7R, DUSP2
unconventional_T_cells	CD3D, KLRB1, FCGR3A, NKG7, GNLY, PTGDS, GZMB, PRF1, KLRD1, GZMA, CCL5
NK-like_cells	GZMK, KLRB1, KLRC1, KLRD1, NKG7, CD69, DUSP2, CD247
NK_cells	KLRC1, GNLY, PRF1
Neutrophils	CEACAM8, FUT4, ITGAM, FCGR3A

Table S3. Primers used for quantitative PCR.

Target	Forward primer (5'-3')	Reverse primer (5'-3')
BCKDK	GCCACAATGGAGAGTCACCTAG	CTTTGTGAGCGATTCCTCCACC
ADCY4	GAGCCTACCTATCTGGTCATCG	GACTCCAGGTAACGGGTCATCA
DENND3	TCGTGAAGGACGCCAGCATCAT	TCCGTCTCCTTCCAGCAGTTGT
ADH4	CCTTGACTGTGCAGGTGGATCT	GTCAATCCTTTGCTACCAGCAGC
DPP4	AAAGGCACCTGGGAAGTCATCG	CAGCTCACAACTGAGGCATGTC

Table S4. Types of organ failure in patients with ACLF (N = 15)

Organ failure	N (%)
Liver failure	10 (66.7)
Kidney failure	9 (60.0)
Cerebral failure	0 (0.0)
Coagulation failure	2 (13.3)
Circulation failure	8 (53.3)
Respiratory failure	4 (26.7)

Data are presented as N (%) of the total ACLF cohort (N = 15).

Table S5. Types of infections in patients according to disease stage

Type of infection	Comp (n=9)	AD (n=15)	ACLF (n=15)	Total (N=39)
Any infection	0 (0.0)	1 (6.7)	11 (73.3)	12 (30.8)
Type of infection				
Spontaneous bacterial peritonitis (SBP)	0 (0.0)	0 (0.0)	3 (20.0)	3 (7.7)
Pneumonia	0 (0.0)	0 (0.0)	2 (13.3)	2 (5.1)
Urinary tract infection	0 (0.0)	0 (0.0)	2 (13.3)	2 (5.1)
Skin and soft tissue infection	0 (0.0)	0 (0.0)	2 (13.3)	2 (5.1)
Intra-abdominal abscess	0 (0.0)	0 (0.0)	1 (6.7)	1 (2.6)
Bacteremia without identified primary focus	0 (0.0)	0 (0.0)	1 (6.7)	1 (2.6)
Suspected infection without confirmed focus	0 (0.0)	1 (6.7)	1 (6.7)	2 (5.1)

Data are presented as N (%). One patient with ACLF had infections at two anatomically distinct sites and is counted in both corresponding categories
