# Supplementary material for: Spatial transcriptomics reveal impaired metabolic liver zonation in acute-on-chronic liver failure
Source: JHEP Rep. 2026 Sep 10;8(12):102035. doi: 10.1016/j.jhepr.2026.102035 (PMC13756263; doi:10.1016/j.jhepr.2026.102035)
Supplement: Supplementary file 3 [file mmc4.pdf]

Spatial transcriptomics reveal impaired metabolic liver zonation in acute-on-chronic liver failure

Authors

Mona-May Langer, Helena Stadler, Mareike Mannewitz, …, Katja Steiger, Bettina Budeus, Christian M. Lange

Correspondence

Christian.Lange@unimedizin-mainz.de (C.M. Lange).

Graphical abstract

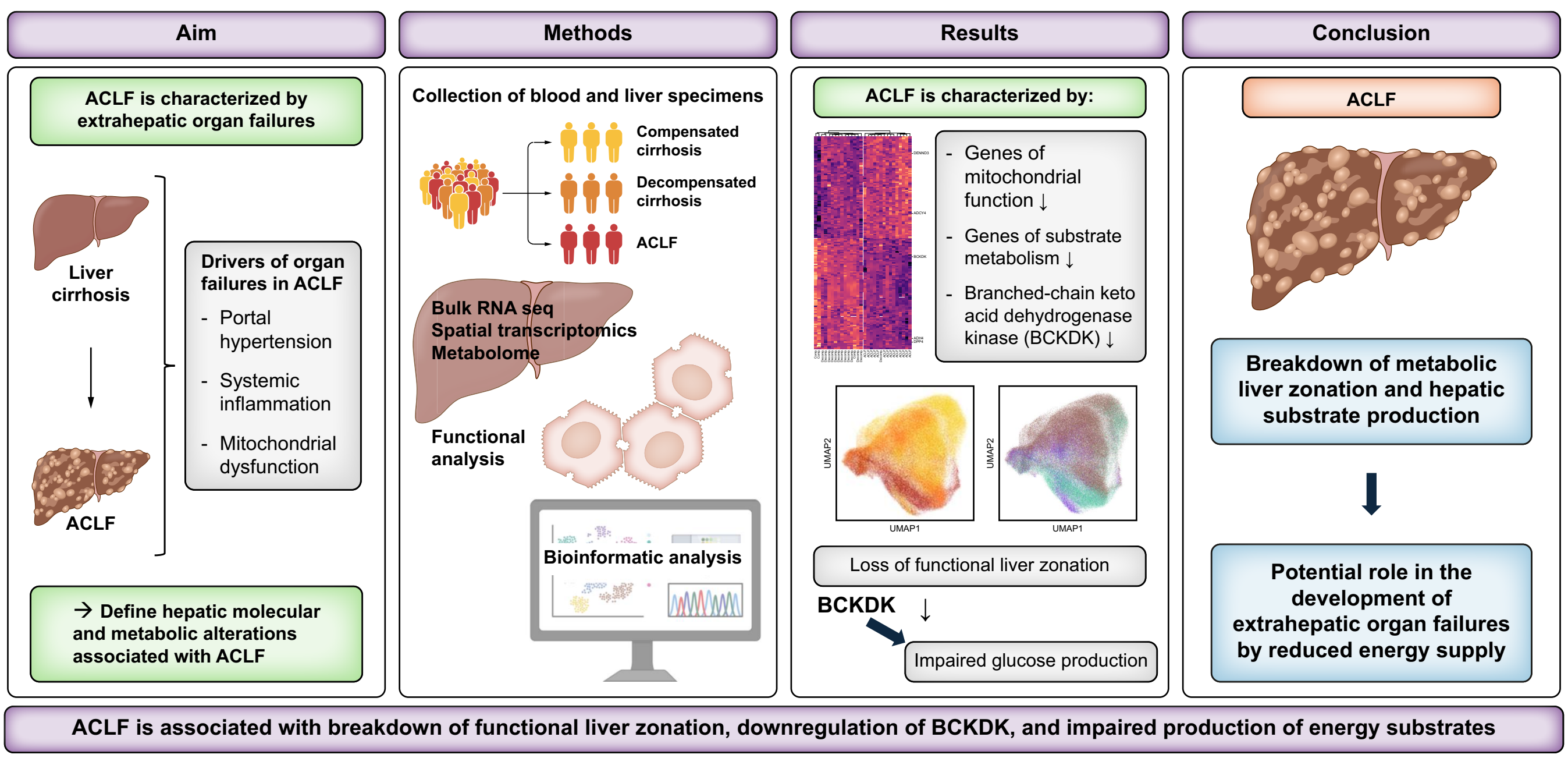

Highlights:

- RNA sequencing and spatial transcriptomics were performed in patients with compensated cirrhosis, decompensated cirrhosis, or ACLF.
- ACLF showed profound hepatic transcriptome changes, clearly distinct from compensated and decompensated cirrhosis.
- Most significant, ACLF was associated with downregulation of pathways involved in substrate metabolism and mitochondrial function.
- In line, spatial transcriptomics revealed a breakdown of functional liver zonation in ACLF.
- Downregulation of BCKDK in ACLF may link dysregulated metabolism of branched-chain amino acids with impaired glucose production.

Impact and implications:

Extrahepatic organ failures are well known features of ACLF, but the contribution of the liver itself to their pathogenesis is not well understood. By RNA sequencing and spatial transcriptomics of liver specimens, we could identify profoundly impaired metabolic and mitochondrial hepatic programs, suggesting that the liver might play a key role in the development of extrahepatic organ failures by compromising the body's energy supply. These findings are important as they provide a rationale for further studying molecular targets in the liver as therapeutic strategies for ACLF.

https://doi.org/10.1016/j.jhepr.2026.102035
© 2026 The Authors. Published by Elsevier B.V. on behalf of European Association for the Study of the Liver (EASL). This is an open access article under the CC BY license (http://creativecommons.org/licenses/by/4.0/).

Spatial transcriptomics reveal impaired metabolic liver zonation in acute-on-chronic liver failure

Mona-May Langer[1], Helena Stadler[1], Mareike Mannewitz[1], Alina Bauschen[1], Severin Jacobi[2], Dionysios Koliogiannis[2], Markus Guba[2], Devon Siemes[3], Daniel R. Engel[3], Carolin Mogler[4], Johannes Wirth[4], Katja Steiger[4], Bettina Budeus[5], Christian M. Lange[1,6,*]

JHEP Reports **2026**. vol. 8 | 1–11

Check for updates

Background & Aims: Extrahepatic organ failures are key determinants of acute-on-chronic liver failure (ACLF). While the role of systemic inflammation and mitochondrial dysfunction in the pathogenesis of ACLF is well known, the contribution of the liver itself is less understood. We therefore performed transcriptome and functional analyses of the liver to identify hepatic drivers of ACLF.

Methods: Bulk RNA sequencing and spatial transcriptomics were performed in liver specimens from patients with compensated cirrhosis, decompensated cirrhosis or ACLF. In addition, detailed analyses of genes involved in energy metabolism, as well as a metabolome analysis and functional analyses were performed.

Results: In total, 39 patients were included (nine compensated cirrhosis, 15 decompensated cirrhosis, and 15 ACLF). ACLF was associated with changes in the hepatic transcriptome, clearly distinct from compensated and decompensated cirrhosis. Most strongly downregulated in ACLF were pathways involved in substrate metabolism and mitochondrial function, including downregulation of multiple genes of glucose and amino acid metabolism. In line, spatial transcriptomics revealed a breakdown of the functional liver zonation in ACLF, with decreasing proportions of hepatocytes specialized in gluconeogenesis/β-oxidation (compensated cirrhosis 68%, decompensated cirrhosis 52%, ACLF 12%; p <0.03). Branched-chain ketoacid dehydrogenase kinase (BCKDK), a key regulator of branched-chain amino acid catabolism, was strongly downregulated in ACLF (0.33-fold expression *vs.* compensated cirrhosis, p <0.05) and may link dysregulation of branched-chain amino acid and glucose metabolism.

Conclusions: ACLF is characterized by distinct changes in the hepatic transcriptome, which affect key pathways in substrate metabolism and mitochondrial function and are associated with loss of functional liver zonation. Downregulation of BCKDK in ACLF might be of particular interest, as it links altered branched-chained amino acid metabolism to impaired glucose production.

© 2026 The Authors. Published by Elsevier B.V. on behalf of European Association for the Study of the Liver (EASL). This is an open access article under the CC BY license (http://creativecommons.org/licenses/by/4.0/).

Introduction

Acute-on-chronic liver failure (ACLF) is a clinical syndrome characterized by acute decompensation (AD) of liver cirrhosis in combination with defined organ failures and high short-term mortality. The most widely accepted definition of ACLF has been established clinically by the European Association for the Study of Liver - Chronic Liver Failure (EASL-CLIF) consortium from the large, prospective CANONIC study.[1] Of note, this study has clearly identified the importance of extrahepatic organ failures as main drivers of short-term mortality of ACLF.[1] Precipitating events of ACLF can be of hepatic and extrahepatic origin, but both types of triggers result in the same clinical syndrome.[2,3] At least in the Western world, infections and alcoholic hepatitis are by far the most frequent triggers of ACLF.[3]

In line with these clinical observations, extensive research has now revealed important pathogenetic processes which contribute to the development of ACLF and its mortality. These include, beyond portal hypertension and liver insufficiency, functionally linked systemic inflammation, (hepatic) cell death, and mitochondrial dysfunction.[2,4–6]

Although the importance of these phenomena as drivers of the syndrome has become very clear, the evidence has mainly been derived from studies evaluating changes in inflammatory mediators, cell death markers and immune cells in blood, from large blood metabolome studies, and from studies investigating mitochondrial function in peripheral immune cells.[2,4–10] In contrast, data investigating the role of the liver itself on a molecular level in the progression from decompensated cirrhosis to ACLF are scarce.

Branched-chain ketoacid dehydrogenase kinase (BCKDK) is a key regulator of branched-chain amino acid metabolism, as it promotes phosphorylation and thereby inactivation of BCKDH, the major enzyme responsible for oxidative decarboxylation of branched-chain amino acids (BCAAs). Hence,

* Corresponding author. Address: Department of Internal Medicine I, University Medical Center of the Johannes Gutenberg University Mainz, Langenbeckstr. 1, D-55131 Mainz, Germany. Tel.: +49 6131 177276; fax: +49 6131 175595.
E-mail address: Christian.Lange@unimedizin-mainz.de (C.M. Lange).
https://doi.org/10.1016/j.jhepr.2026.102035

inhibition or downregulation of BCKDK results in reduced phosphorylation of branched-chain α-ketoacid dehydrogenase (BCKDH) and higher rates of BCAA catabolism. Furthermore, BCKDK has been identified as an important regulator of glucose metabolism by multiple mechanisms, dependent and independent from BCAA concentrations.[11–13]

In the present study, we characterized changes in the hepatic transcriptome in compensated or decompensated cirrhosis and ACLF on a global and spatial level, coupled with adjunctive functional analyses, and identify severe hepatic metabolic dysfunction and loss of BCKDK as a potential key driver of this process.

Patients and methods

Patients

All consecutive patients with liver cirrhosis who underwent a liver transplantation at the LMU university Hospital Munich between October 2021 and December 2024 were included, if they agreed to participate in the study and if prompt processing of collected biospecimens could be ensured. Patients with active alcohol consumption during the last 6 months, pregnant and breast-feeding patients, as well as patients with hepatocellular carcinoma beyond Milan criteria or patients tested positive for human immunodeficiency virus were excluded from the study. ACLF was classified according to EASL-CLIF-criteria directly.[1] Grade of ACLF and types of organ failures were not included as selection criteria for the study. Patients with infections before liver transplantation were only included, if infections were well controlled with antibiotics. Disease stage was classified at the time of liver transplantation (*i.e.* at the time of tissue harvesting). Written informed consent was obtained from participants, and the study was approved by the local ethical committee (LMU 22-0392).

Liver biopsies, RNA isolation and sequencing

Liver biopsies from explant liver were obtained during liver transplantation directly after hepatectomy. For later use, a portion of the biopsies was immediately snap-frozen in liquid nitrogen and stored at -80°C, while another portion was preserved in RNAlater (Invitrogen, Waltham, MA, USA) at -20°C for subsequent RNA isolation. Total RNA was extracted using the RNeasy Mini Kit (Qiagen, Hilden, Germany) according to the manufacturer's instructions. Concentration and quality of the RNA were measured with a Qubit (Invitrogen, Waltham, MA, USA) and an Agilent Bioanalyzer DNA HS (Agilent, Santa Clara, CA, USA). Library preparation was performed with a Lexogens QuantSeq 3′ mRNA-Seq Library Prep Kit FWD and sequenced on a NextSeq500 (Illumina, San Diego, CA, USA). Adapter sequences were removed with TrimGalore,[14] and sequences were aligned with DESeq2.[15] RNA sequencing (RNA-seq) data have been deposited at Gene Expression Omnibus (GEO) and are publicly available as of the date of publication (accession number: GSE322724).

Gene expression data processing and analysis of bulk RNA-seq

Statistical analysis was performed with R (version 4.5.1, R Foundation for Statistical Computing, Vienna, Austria). Bulk RNA-seq data were processed using DESeq2.[15] Heatmaps and Upset Plots were created with ComplexHeatmaps (version 2.18.0).[16] Gene set enrichment analysis (GSEA) was performed with fgsea (10.1101/060012) and volcano plots with EnhancedVolcano (version 1.14.0).[17]

Single-cell spatial transcriptomics using Xenium In Situ

Spatial transcriptomics was performed in a subgroup of selected patients with compensated cirrhosis, decompensated cirrhosis, or ACLF (n = 3, each). Selection criteria for spatial transcriptomics were: evidence of high quality of the histologic specimen based on analyses of H&E staining by an expert pathologist (CM), absence of ongoing infections, absence of ongoing viral replication (in case of viral hepatitis), absence of cholestatic/autoimmune diseases as underlying etiologies to exclude possible bias. Among patients who fulfilled these criteria, we randomly selected three patients per group. Then, Xenium *In Situ* (10X Genomics, Pleasanton, United States) experiments of frozen samples were performed using a custom Xenium panel with 477 genes (Table S1). Tissue sectioning and sample preprocessing were performed according to the manufacturer's protocol. Briefly, 10 μm sections of the frozen samples were prepared at -20°C using a cryotome and mounted on Xenium slide. A finger was pressed against the back of the slide until the section adhered completely. After short-term storage at -80°C, the samples went through a 3-day protocol including multiple steps such as paraformaldehyde fixation, permeabilization, probe hybridization, ligation, amplification, and cell segmentation staining. The preprocessed samples were analyzed using the Xenium Analyzer with the instrument software version 4.0.1.4 and analysis version 4.0.1.0. Subsequently, H&E staining of the tissue sections was performed according to the manufacturer's protocol for post-Xenium staining.

Spatial gene expression analysis

Spatial transcriptomics data were processed and analyzed in Python (version 3.9.21, Python Software Foundation, Beaverton, OR, USA) using stLearn (version 0.4.12)[18] and Scanpy (version 1.9.8).[19] After merging samples, low-quality genes and cells (<10 total counts) were filtered out, followed by normalization and log transformation. Dimensional reduction was performed using principal component analysis (PCA), followed by construction of a k-nearest neighbor graph, Louvain clustering, and uniform manifold approximation and projection (UMAP) visualization. Batch correction across experimental groups was performed using BBKNN (version 1.6.0).[20] Cell-type annotation was done by scoring expression of marker genes curated from literature (Table S2).[21]

Cells were classified according to highest marker gene score, and unassigned cells were labeled accordingly. Gene expression patterns across predicted cell types were observed using dot plots. All analyses were performed using NumPy (version 1.20.3),[22] Pandas (version 1.5.2),[23] SciPy (version 1.10.1),[24] and AnnData (version 0.10.5.post1).[25] Figures were generated using Seaborn (version 0.12.2)[26] and Matplotlib.[27] To assess changes in functional hepatocyte subtypes, the number of cells annotated to each subtype was normalized to the total number of hepatocytes per sample.

Quantitative PCR

RNA was isolated as described above and RNA concentration was measured with a NanoDrop™ 2000 Spectrophotometer

(Thermo Fisher Scientific Inc., Waltham, MA, USA). cDNA production and quantitative real-time PCR were performed with the PrimeScript™ RT Reagent Kit and TB Green® Premix Ex Taq™ (Takara Bio Inc., Kusatsu, Japan), according to the manufacturer's instructions. Primer sequences were obtained from OriGene Technologies (Rockville, MD, USA) based on publicly available primer sequences provide by the manufacturer and synthesized by Eurofins Genomics (Eurofins Genomics, Ebersberg, Germany). Primer sequences are shown in Table S3.

Western blot

Proteins were extracted from tissue by homogenization of biopsy samples in cold RIPA buffer (Cell Signaling Technology, Danvers, MA, USA) supplemented with protease and phosphatase inhibitors (cOmplete™, Mini, EDTA-free Protease Inhibitor Cocktail and PhosSTOP™ Phosphatase Inhibitor Cocktail Tablets, Roche Diagnostics GmbH, Mannheim, Germany). Homogenates were incubated at 4°C for 2 h with gentle shaking, followed by centrifugation at 16,000×*g* for 20 min at 4°C. Supernatants containing soluble proteins were stored at -20°C until further usage. Protein concentrations were determined using a Bradford assay (Bio-Rad Laboratories, Hercules, CA, USA). Proteins were separated using a 12% SDS-PAGE and transferred to a Immobilon-P PVDF membrane (Millipore, Billerica, MA, USA). Membranes were blocked with 5% non-fat milk and incubated with primary antibodies at 4°C overnight, followed by incubation with HRP-conjugated secondary antibodies for 1 h at room temperature. Protein bands were visualized using Immobilon Forte Western HRP substrate (Merck KGaA, Darmstadt, Germany). After detection, antibodies were stripped using ReBlot Plus Strong Antibody Stripping Solution (Merck KGaA, Darmstadt, Germany) for 15 min, re-blocked and incubated with a new primary antibody. The following antibodies were used for the study: Phospho-BCKDH-E1α (Ser293) (E2V6B), BCKDH-E1α (E4T3D), and BCKDK (E5B6J) were purchased from Cell Signaling Technology. The Anti-β-Actin (AC-15) antibody was purchased from Merck KGaA and the secondary antibodies Mouse anti-rabbit IgG-HRP and Goat anti-mouse IgG-AP were purchased from Santa Cruz Biotechnology (Dallas, TX, USA). All antibodies were used following the manufacturer's instructions.

Metabolome analysis

Serum and liver tissue samples were subjected to untargeted and targeted metabolomics at the VIB Metabolomics Expertise Center (MEC, Leuven, Belgium). Metabolites were extracted using an 80:20 methanol/water solution. For unbiased profiling and broad coverage, untargeted metabolomic analysis (SCI-MAX) of samples was performed. Samples were subjected to a two-phase extraction, yielding polar and apolar fractions, which were analyzed separately. Metabolite features were detected based on matching *m/z* values and isotope pattern information, followed by annotation through database matching. All potential annotations for each detected *m/z* were provided for the respective polar and apolar fractions. For further verification and accuracy, metabolites involved in BCKDK pathway (metabolites of glycolysis as well as BCAAs) were analyzed by targeted metabolomics allowing quantitative measurement. Samples were analyzed by ion-pairing liquid chromatography-mass spectrometry. Metabolite abundances

Table 1. Baseline characteristics and laboratory results of included patients.

	Comp. (n = 9)	AD (n = 15)	ACLF (n = 15)	*p* value group comparison	*p* value (Comp. *vs.* AD)	*p* value (Comp. *vs.* ACLF)	*p* value (AD *vs.* ACLF)
Age (years), mean (SD)	57.9 (5.1)	53.2 (10.4)	54.5 (8.4)	0.453	–	–	–
Female sex, n (%)	1 (11.1)	4 (26.6)	6 (40.0)	0.354	–	–	–
CLIF-OF score, mean (SD)	6.7 (1.0)	7.5 (1.1)	10.1 (2.0)	<0.001	0.693	0.009	<0.001
MELD score, mean (SD)	9.9 (2.8)	18.0 (6.2)	30.5 (5.6)	<0.001	0.119	<0.001	0.001
Etiology of cirrhosis (multiple etiologies per patient possible)							
Viral, n (%)	3 (33.3)	0 (0.0)	0 (0.0)	0.009	0.126	0.126	–
MASLD, n (%)	0 (0.0)	2 (13.3)	1 (6.7)	0.778	–	–	–
Alcoholic, n (%)	5 (55.6)	8 (53.3)	10 (66.7)	0.773	–	–	–
Others, n (%)	2 (22.2)	6 (40.0)	5 (3.3)	0.765	–	–	–
Leukocytes (10^9/L), mean (SD)	5.2 (2.5)	4.5 (2.3)	11.3 (6.7)	0.002	1.0	0.076	0.002
Hemoglobin (g/dl), mean (SD)	12.8 (0.9)	9.0 (2.3)	8.0 (1.0)	<0.001	0.003	<0.001	0.938
Platelets (10^9/L), mean (SD)	105.6 (42.7)	89.8 (54.5)	78.8 (71.3)	0.183	–	–	–
CRP (mg/dl), mean (SD)	0.5 (0.4)	1.4 (1.1)	2.9 (3.2)	0.008	0.207	0.006	0.436
Creatinine (mg/dl), mean (SD)	0.9 (0.2)	1.0 (0.4)	2.4 (1.0)	<0.001	1.0	<0.001	<0.001
Bilirubin (mg/dl), mean (SD)	1.1 (0.8)	7.2 (8.3)	17.8 (13.6)	<0.001	0.060	<0.001	0.151
ALT (U/L), mean (SD)	31.4 (9.7)	36.2 (26.7)	60.0 (65.8)	0.568	–	–	–
INR, mean (SD)	1.3 (0.2)	1.8 (0.63)	2.1 (0.5)	<0.001	0.032	<0.001	0.201
ACLF grade				–	–	–	–
1, n (%)	–	–	5 (33.3)				
2, n (%)	–	–	5 (33.3)				
3, n (%)	–	–	5 (33.3)				
Hepatic encephalopathy, n (%)	0 (0.0)	1 (6.7)	5 (33.3)	0.089	–	–	–
Gastrointestinal bleeding, n (%)	0 (0.0)	0 (0.0)	2 (13.3)	0.332	–	–	–
Infections, n (%)	0 (0.0)	1 (6.7)	11 (73.3)	<0.001	1.0	0.002	0.001
Ascites, n (%)	0 (0)	10 (66.7)	14 (93.3)	<0.001	0.078	0.001	0.666

p values were calculated with the Kruskal-Wallis test for metric variables followed by pairwise comparisons using the Mann-Whitney *U* test with Bonferroni-correction. Differences in categorical variables among Compensated, AD, and ACLF were evaluated using Fisher's exact test for R×C tables. If the overall test was significant, pairwise 2×2 Fisher's exact tests were performed (Comp. *vs.* AD, Comp. *vs.* ACLF, AD *vs.* ACLF). The *p* values are Bonferroni-corrected for three pairwise group comparisons (Comp. *vs.* AD, Comp. *vs.* ACLF, AD *vs.* ACLF). ALT, alanine aminotransferase; CLIF-OF, chronic liver failure–organ failure; CRP, C-reactive protein; INR, international normalized ratio; MELD, model of end-stage liver disease, MASLD, metabolic dysfunction-associated steatotic liver disease.

were reported as integrated peak areas (raw abundances) and detection limits were determined based on blank samples.

Glucose production assay

To assess functional implications of downregulated BCKDK on glucose metabolism, a glucose production assay was performed. To this end, cryopreserved human primary hepatocytes (Lonza, Basel, Switzerland) were used and plated according to manufacturer`s instructions and seeded on collagen-coated 12-well plates at 2×10^5 cells per well in Maintenance Medium (Lonza). After plating for 24 h, cells were serum-starved in DMEM with 3.7 g/L sodium bicarbonate (Pan Biotech, Aidenbach, Germany) supplemented with 1 g/L glucose (Merck KGaA) and 4 mM L-glutamine (Pan Biotech) for 16 h. BT2 (MedChemExpress, Monmouth Junction, NJ, USA; dissolved in DMSO), a BCKDK-inhibitor, was added to serum-starving-media to achieve final concentrations of 80 µM or 200 µM. Positive and negative controls received equivalent DMSO concentrations. Following starvation phase, cells were extensively washed with PBS and incubated for up to 24 h in 0.3 ml glucose-free DMEM with 3.7 g/L sodium bicarbonate supplemented with 2 mM glutamine, 15 mM HEPES, 20 mM lactate, 2 mM pyruvate and 0.1 mM pCPT-cAMP (all Merck KGaA). Negative controls received medium without pCPT-cAMP. Supernatants were collected at indicated time points (3 h, 6 h, and 24 h) and glucose detection was performed with Invitrogen Glucose Colorimetric Detection kit (Thermo Fisher Scientific Inc.; #EIAGLUC), according to manufacturer's protocol and the 4PL regression model was used for calculations.

Statistical analysis

Statistical analysis was performed using GraphPad Prism version 9.0.2 software (GraphPad Software, San Diego, CA, USA). After testing for Gaussian distribution, two groups were

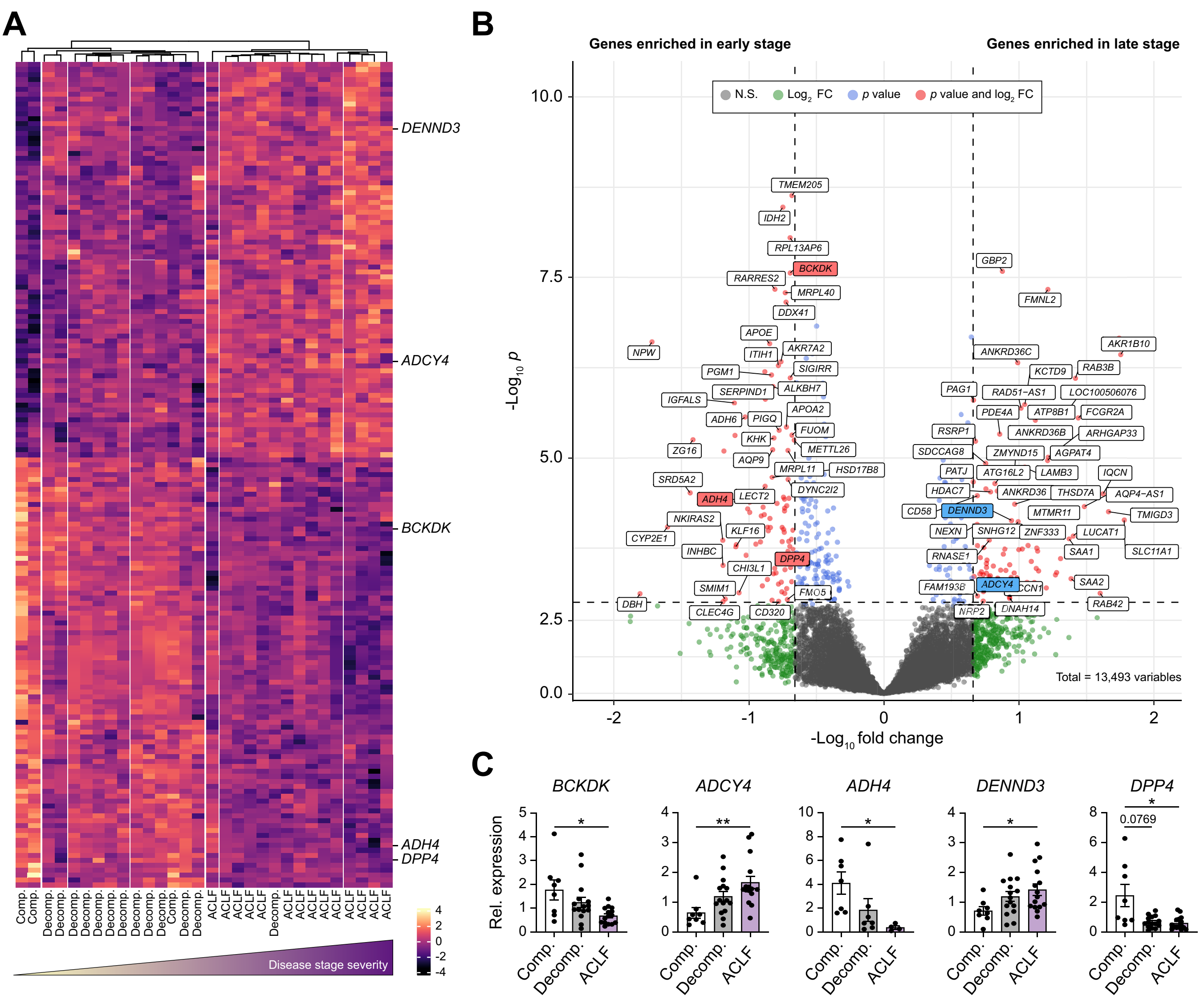

Fig. 1. ACLF is associated with distinct changes of the hepatic transcriptome. (A) Heatmap showing unsupervised clustering of the top differentially regulated genes (DRGs) across stages of liver cirrhosis (p_{adj} <0.03 and abs(log_2FC) >0.65). Genes confirmed in qPCR are noted. For a full list of genes, please see Fig. S1. (B) Volcano plot of all differentially expressed genes across stages of liver cirrhosis. (C) Quantification of expression of selected genes by real-time PCR. ACLF, acute-on-chronic liver failure; comp., compensated cirrhosis; decomp., decompensated cirrhosis.

compared using a *t* test or Wilcoxon-Mann-Whitney *U* test, as appropriate, while comparison of more groups was done using one-way ANOVA or the Kruskal-Wallis test, as appropriate. Values of p <0.05 were considered to be statistically significant.

Results

Patient characteristics

A total of 39 patients were included in the present study, of whom nine (23.1%) had compensated cirrhosis, 15 (38.5%) had decompensated cirrhosis, and 15 (38.5%) had ACLF, respectively. Detailed baseline characteristics are shown in Table 1. Types of organ failures in ACLF and data on infections are shown in Table S4 and S5.

ACLF is associated with impaired expression of genes involved in energy metabolism and mitochondrial function

Gene expression of liver specimens was first assessed by bulk RNA-seq to understand whether ACLF is associated with distinct changes in the hepatic transcriptome. For this analysis, liver specimens of the first consecutive 30 patients of the entire cohort were used (three compensated cirrhosis, 13 decompensated cirrhosis, and 14 ACLF). Of note, unsupervised clustering revealed a distinct hepatic transcriptome in ACLF compared with compensated and decompensated cirrhosis (Fig. 1A and Fig. S1). As shown in Fig. 1A,B, the most strongly regulated genes in ACLF compared to compensated/decompensated cirrhosis included genes involved in substrate metabolism and regulation of mitochondrial function, namely

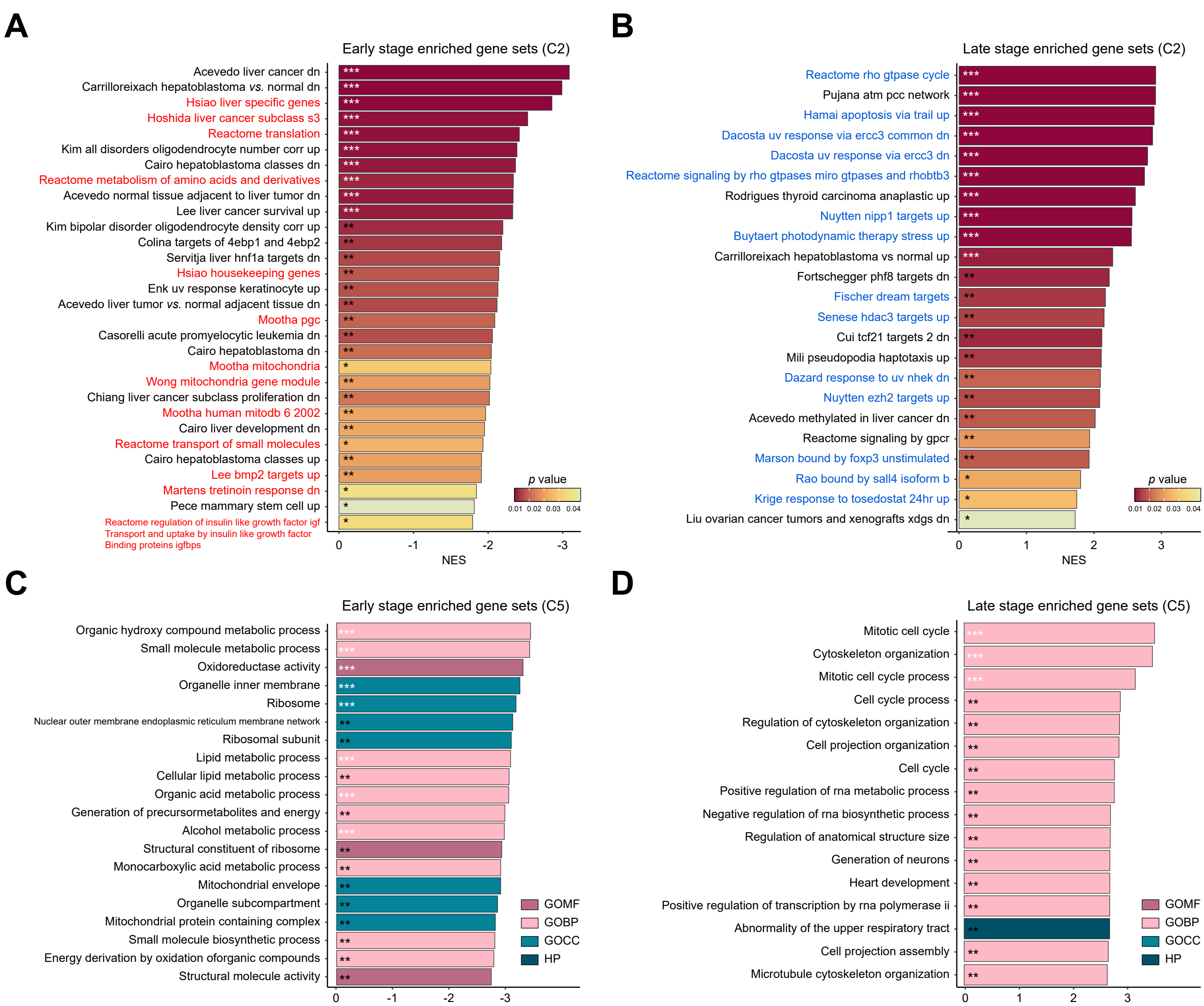

Fig. 2. ACLF is associated with dysregulation of pathways involved in metabolism and mitochondrial function. (A,B) Gene set enrichment analysis results for C2-curated gene sets and (C,D) C5-GO terms separated in gene sets enriched in early stages and late stages of liver cirrhosis. Genes with p_{adj} <0.05 were selected for the gene set enrichment analysis. Gene sets marked in red are associated with energy metabolism. Gene sets marked in blue are associated with poor DNA repair and proliferative capacity. GO terms: GOMF (magenta), molecular function; GOBP (pink), biological process; GOCC (blue), cellular component; HP, human phenotypes.

BCKDK, ADCY4, DPP4, and ADH4, as well as in autophagy as a downstream event of substrate shortage (DENND3). For further validation, expression of selected top genes was quantified by qPCR in liver specimens of all patients included in this study (*i.e.* all patients in whom RNA-seq was performed and eight subsequent patients), Fig. 1C. Expression of BCKDK was 0.33- and 0.5-fold compared with compensated and decompensated cirrhosis, respectively (p <0.05). Of note, expression of these genes correlated only moderately with markers of systemic inflammation (Fig. S2) and no strong influence of infections or the type of organ failures on the expression of these genes was observed (Fig. S3 and S4).

Next, gene enrichment analysis of genes which were differentially regulated over disease stage severity was performed (Fig. 2). Among the gene sets from C2 (curated gene sets) and from C5–GO, enriched in early stages are gene sets depicting normal liver cells and gene sets involved in in energy metabolism, amino acid metabolism and mitochondrial function (Fig. 2A,C). The gene sets enriched in late stages (ACLF) suggest a disturbed DNA repair and cell proliferation (*e.g.* NIPP1 upregulation,[28] response to UV or response to tosedostat[29,30]). Taken together ACLF shows an RNA expression profile with reduced mitochondrial function, energy metabolism including amino acid metabolism, DNA repair and cell proliferation (Fig. 2B,D).

Spatial resolution of expression of key genes involved in metabolism

Because in the healthy liver expression of genes involved in substrate metabolism is characterized by a strong zonal organization, spatial transcriptomics of liver specimens of nine

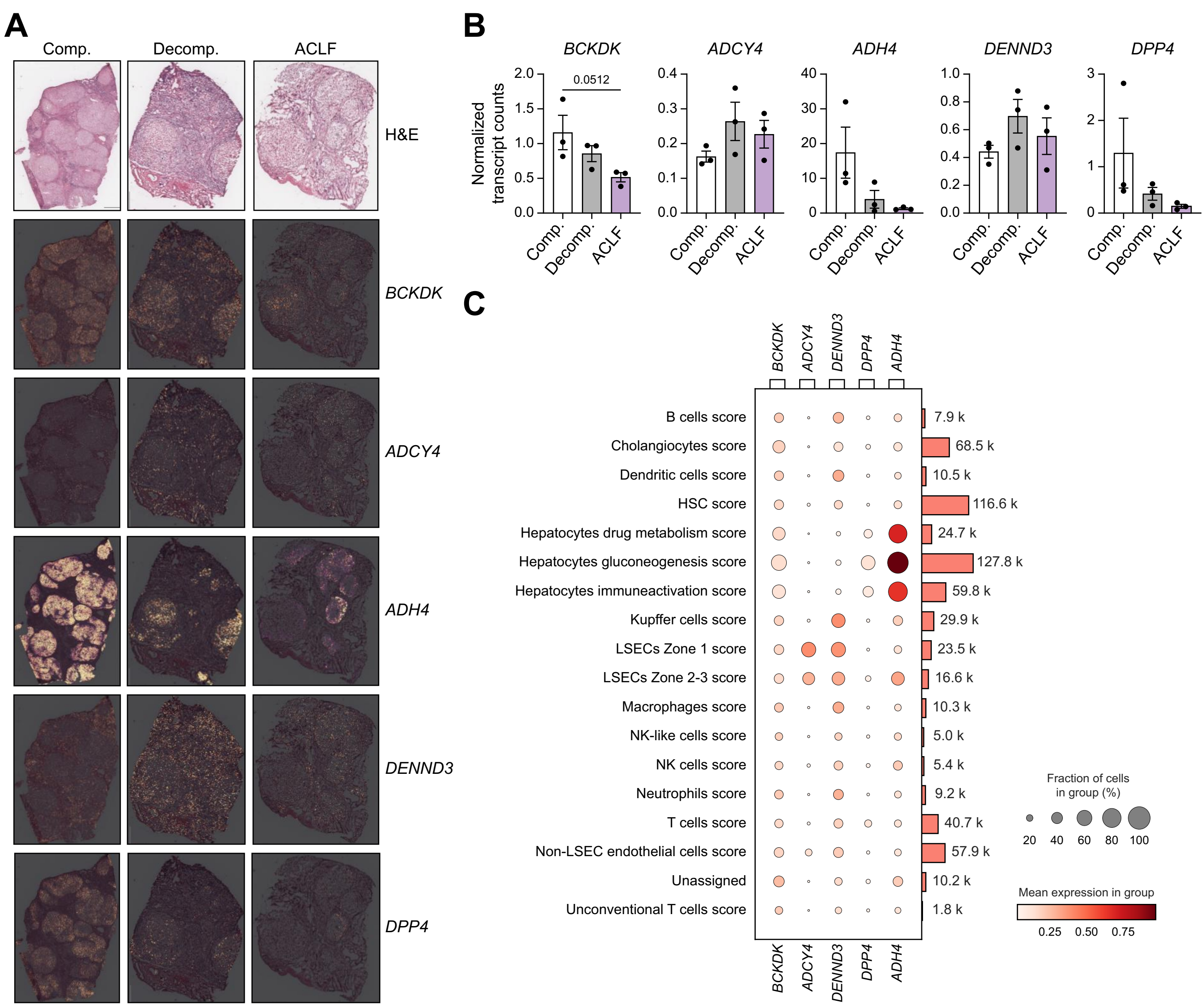

Fig. 3. Spatial distribution of gene expression of selected genes. (A) Representative images of spatial gene expression of top genes identified by bulk RNA sequencing according to the stage of liver cirrhosis. (B) Summary of gene expression of all nine patients included in spatial gene expression analyses. Transcript counts normalized to total cell count per sample are shown. (C) Expression of top hits across predicted cell types. Dot size represents the fraction of cells expressing the gene, and color intensity represents average expression. Totals per cell type are included. Cell types are defined by expression of canonical cell-type markers.[21] ACLF, acute-on-chronic liver failure; comp., compensated cirrhosis; decomp., decompensated cirrhosis.

selected patients (three compensated cirrhosis, three decompensated cirrhosis, and three ACLF, see Methods for selection criteria) was performed to understand whether decompensated cirrhosis/ACLF are associated with changes of functional liver zonation. Spatial expression of key candidate genes (*BCKDK*, *ADCY4*, *ADH4*, *DENND3*, and *DPP4*) is shown in Fig. 3A,B, and overall supports the findings of bulk RNA-seq. Spatial transcriptomics further revealed that BCKDK (and DPP4) was particularly expressed in hepatocytes specialized in glucose metabolism, as well as in cholangiocytes, non-Liver sinusoidal endothelial cell (LSEC)-endothelial cells, hepatic stellate cells, and some immune cells (Fig. 3C). In contrast, expression of ADH4 and ADCY4 was largely restricted to hepatocytes and LSECs, respectively (Fig. 3C). DENND3 was broadly expressed in non-hepatocellular liver cells and particularly strong in Kupffer cells and LSECs (Fig. 3C).

ACLF is characterized by changes in the functional zonation of the liver

In addition to the above-described spatial resolution of candidate gene expression, we assessed spatial gene expression analyses for general changes in the metabolic zonation of the liver, using expression of published marker genes.[21] Indeed, ACLF was associated with profound changes in the functional liver zonation with declining frequencies of hepatocytes specialized for gluconeogenesis and β-oxidation in ACLF compared with compensated/decompensated

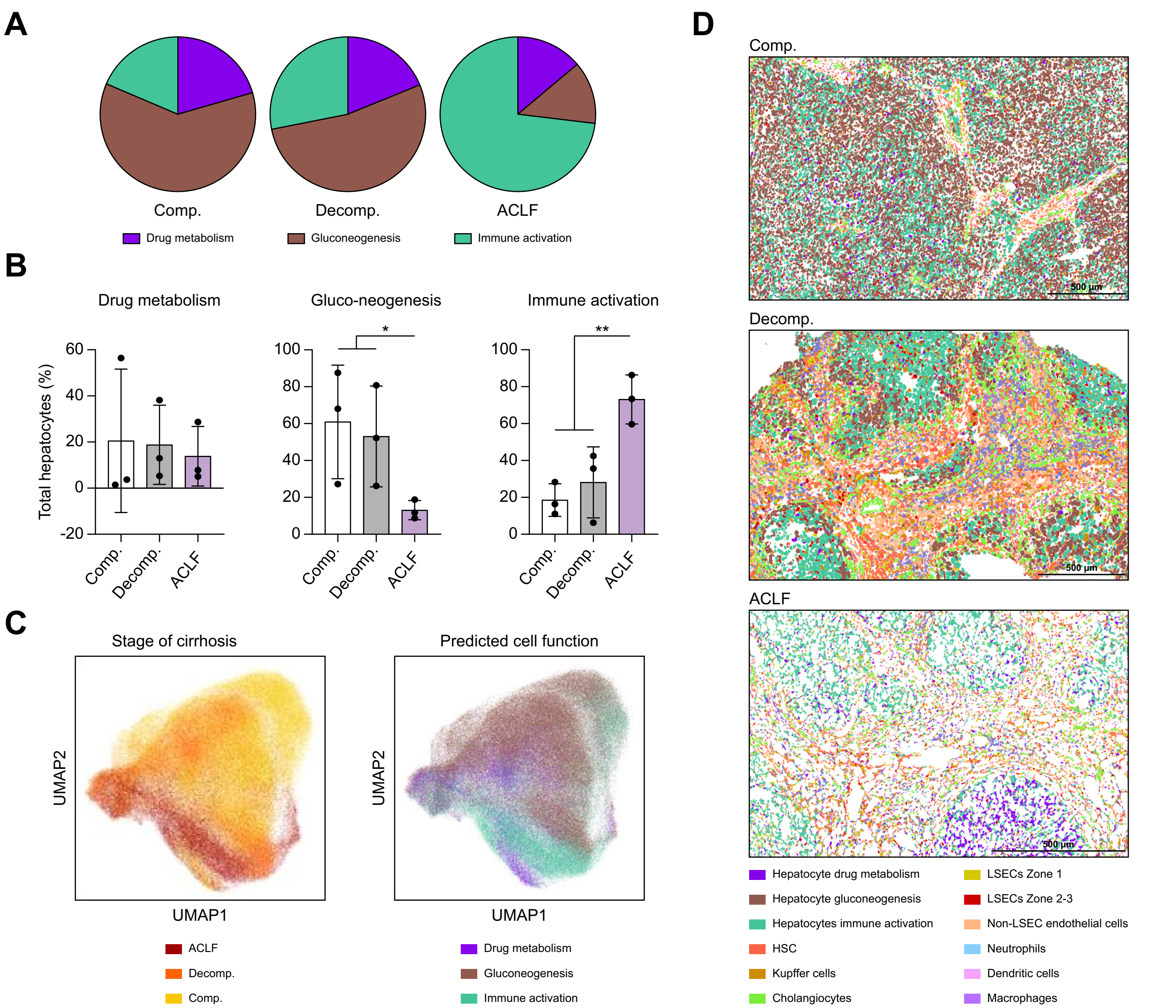

Fig. 4. Anatomical and functional liver zonation according to the stage of liver cirrhosis. (A,B) Metabolic liver zonation, assessed by spatial gene expression analyses of marker genes for drug metabolism, gluconeogenesis/β-oxidation, and immune activation.[21] Relative abundance of functional cell types relative to all hepatocytes is shown. (C) UMAP analyses of differentially regulated genes according to the stage of cirrhosis (left) and to the predicted function of hepatocytes (right), relative to all hepatocytes. (D) Representative images of cell clusters, based on the expression of marker genes, according to the stage of cirrhosis. ACLF, acute-on-chronic liver failure; comp., compensated cirrhosis; decomp., decompensated cirrhosis; UMAP, uniform manifold approximation and projection.

cirrhosis and increasing frequencies of hepatocytes associated with immune activation in ACLF (Fig. 4).

ACLF is associated with dysregulation of multiple genes involved in glucose, fructose, and fatty acid metabolism

In addition to the above-described unsupervised analysis, we specifically explored our bulk RNA-seq dataset for the expression of key genes of glucose and fatty acid metabolism. Of note, the expression of multiple genes involved in glycolysis (PGM1, PGAM1, PKLR, and DLD), gluconeogenesis (PGAM1) and fructose catabolism (KHK) were downregulated in ACLF (Fig. 5A). Furthermore, key genes in fatty acid biosynthesis and β-oxidation (ACSL5 and HSD17B8) were downregulated in ACLF (Fig. 5B).

BCKDK expression is associated with impaired glucose metabolism

BCKDK, one of the top hits of our analysis, is a key regulator of BCAA metabolism. BCKDK phosphorylates and inactivates BCKDH, the key enzyme of BCAAs catabolism (details above). Hence, reduced BCKDK should result in reduced phosphorylation of BCKDH and higher rates of BCAA catabolism (Fig. 6E). Indeed, p-BCKDH concentrations in ACLF were lower compared with compensated/decompensated cirrhosis (Fig. 6A). Of note, BCKDK expression correlates with multiple genes involved in glucose metabolism (Fig. 6B). As described above, BCKDK serves as an important regulator of glucose metabolism by multiple mechanisms, including impaired hepatic production of glucose and downregulation of genes involved in gluconeogenesis, promotion of glycolysis, and inhibition of the pyruvate dehydrogenase complex.[11–13] Hence, we further assessed whether BCKDK is associated with glucose concentrations in blood and metabolites of glucose metabolism in liver tissue. Indeed, ACLF was associated with lower blood glucose concentrations, and hepatic BCKDK expression correlated with pyruvate serum concentration and inversely with blood glucose concentration (Fig. 6C). Moreover, BCKDK was associated with hepatic hexose inositol phosphate and hexose bisphosphate concentrations, representing key intermediates in glycolysis (Fig. 6D). To further support a causal role of impaired BCKDK activity on hepatic glucose metabolism, a glucose production assay was performed. To this end, primary hepatocytes were treated with a synthetic inhibitor (BT2) of BCKDK. Indeed, treatment with BT2 significantly reduced glucose production in primary hepatocytes (Fig. 6E). A sketch summarizing the putative role of BCKDK in ACLF is shown in Fig. 6F.

Discussion

In the present study, we show, by unsupervised clustering of RNA-seq data, that ACLF is characterized by distinct changes in the hepatic transcriptome, which are clearly different from the hepatic transcriptome of compensated and decompensated cirrhosis. This observation is important since the EASL-CLIF definition of ACLF, which was also applied in the

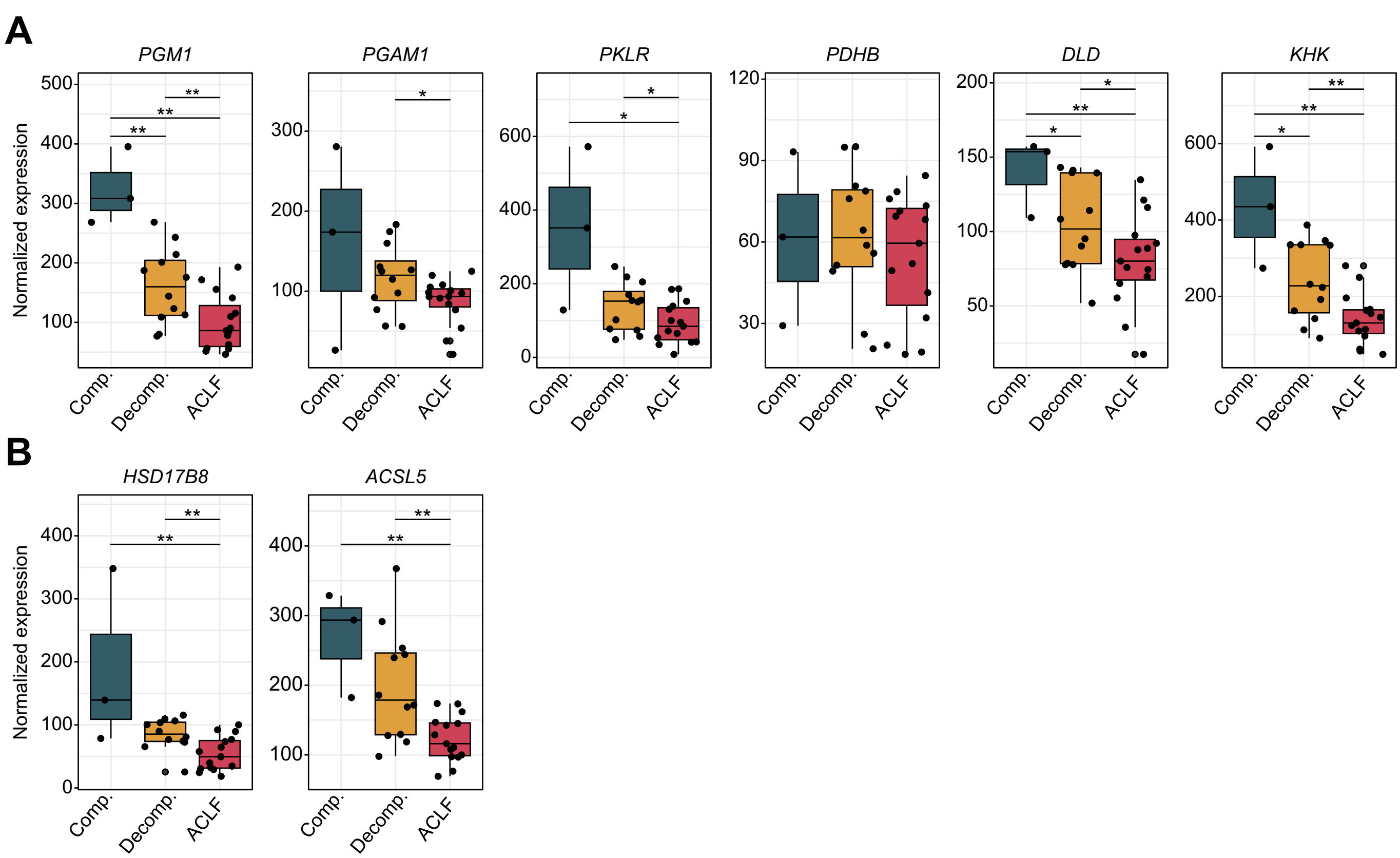

Fig. 5. ACLF is associated with impaired expression of genes involved in metabolism of glucose, fructose and fatty acids. Normalized expression of selected genes from bulk RNA-seq involved in glucose and fructose metabolism (A), as well as in fatty acid/ethanol metabolism (B) from bulk RNA sequencing. Mann-Whitney *U* test. *p ≤0.05, **p ≤0.01, ***p ≤0.001. ACLF, acute-on-chronic liver failure; comp., compensated cirrhosis; decomp., decompensated cirrhosis.

present study, focuses on extrahepatic organ failures to define ACLF, while hepatic failure itself is not central in its definition. The EASL-CLIF definition has been derived from clinical data with the purpose of identifying those patients with AD of cirrhosis that are at high risk of short-term mortality.[1] Without doubt, this definition is of high clinical value and the prognostic relevance of ACLF and associated extrahepatic organ failures has been proven by numerous subsequent studies. In this regard, our study does not question the relevance of extra-hepatic organ failures in the clinical syndrome of ACLF, but our identification of strong hepatic transcriptome changes in ACLF might be important for a better understanding of the patho-physiology of ACLF.

In general, systemic inflammation and mitochondrial dysfunction are considered as key determinants of the development of ACLF-defining organ failures.[4,5,7,9] Yet, the detailed involvement of the liver itself in inflammation and mitochondrial dysfunction is not well understood. Our study points to a profoundly impaired metabolic function of the liver in ACLF, as pathways of substrate metabolism, including amino acid metabolism, and mitochondrial function are strongly down-regulated in ACLF. In line with this observation, spatial transcriptomics have revealed a clear loss of the functional zonation of the liver in ACLF. In particular, there was an evident functional loss of hepatocytes specialized in gluconeogenesis and β-oxidation in ACLF. Given the key role of the liver in the body's supply with glucose and other substrates, it appears plausible that a breakdown of the liver's capacity to control the key underlying metabolic processes contributes to extrahepatic organ failures.

Although certainly multiple genes involved in substrate metabolism and mitochondrial function are dysregulated in ACLF, we have identified BCKDK among the most strongly affected genes, a candidate which might link a breakdown of the hepatic BCAA metabolism with impaired glucose metabolism. A downregulation of BCKDK in ACLF was observed in

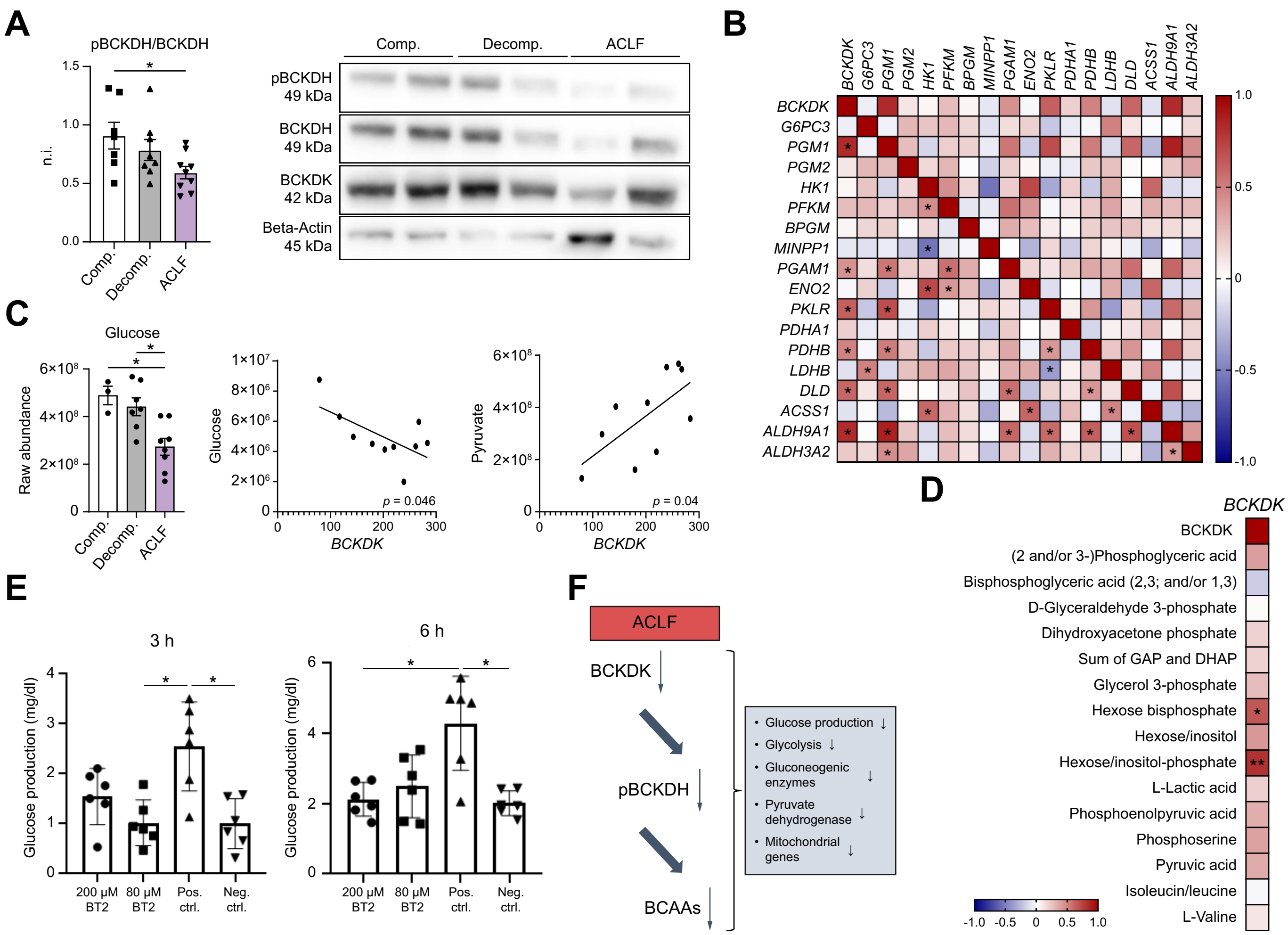

Fig. 6. BCKDK is linked to impaired glucose metabolism in ACLF. (A) Western blot analysis of p-BCKDH in liver specimens from patients with compensated cirrhosis, decompensated cirrhosis or ACLF. All patients with available protein samples were included in the analyses (left side), while a representative gel is shown on the right side. (B) Correlation matrix of BCKDK and selected genes involved in glucose metabolism from bulk RNA sequencing. (C) Serum glucose concentrations according to the stage of liver cirrhosis (left) and correlations between hepatic BCKDK expression and serum glucose and pyruvate concentration. (D) Correlation matrix showing pairwise Pearson correlations between hepatic BCKDK mRNA expression and metabolite raw abundance (integrated peak area) measured by targeted IP-LC-MS in liver tissue. Color intensity reflects the strength and direction of the correlation. (E) Glucose production of primary hepatocytes treated with the BCKDK-inhibitor BT2. Negative control received medium without pCPT-cAMP. (F) Scheme of putative consequences of downregulated BCKDK expression in ACLF.[11–13,31,32] ACLF, acute-on-chronic liver failure; BCKDK, branched-chain ketoacid dehydrogenase kinase; comp., compensated cirrhosis; decomp., decompensated cirrhosis.

our study by bulk RNA-seq, spatial transcriptomics and quantitative PCR. Downregulation of BCKDK results in reduced phosphorylation (*i.e.* reduced inhibition) of BCKDH, the key enzyme responsible for degradation of BCAA. Hence, the observed changes should result in higher rates of intrahepatic degradation of BCAA in ACLF. In addition, BCKDK plays an important, pleiotropic role in the regulation of glucose metabolism, and BCKDK downregulation has been shown to suppress hepatic gluconeogenesis, increase glycolysis, inhibit pyruvate dehydrogenase and repress genes involved in gluconeogenesis.[11–13] In line, our study provides evidence that hepatic downregulation of BCKDK in ACLF may contribute to impaired glucose production in the liver, which may potentially aggravate extrahepatic organ failures. Moreover, downregulation of BCKDK has been shown to promote mitochondrial dysfunction by suppressing key metabolic mitochondrial genes.[31,32]

In addition to the described changes in genes involved in metabolism and mitochondrial function, our spatial transcriptomic analysis revealed a strong upregulation of genes involved in immune activation in ACLF. This finding is in line with the well-described systemic inflammation hypothesis as a driver of ACLF. Yet, a detailed description of these changes is beyond the scope of the present manuscript.

Our study has important limitations. Most importantly, the number of patients is relatively small, in particular in the analysis of spatial transcriptomics and – throughout all analyses – of patients with compensated cirrhosis. The latter is explained by the fact that liver transplantations in patients with compensated cirrhosis are rarely performed in our institution. Because of the biological heterogeneity of cirrhosis and ACLF, further studies with larger sample sizes might be necessary to confirm our findings. In addition, the sample size of our study is too small for a definite exploration of differences of the hepatic transcriptome according to the grade of ACLF and type of organ failures, as well as of the possible role of infections as drivers of the observed differences in ACLF. In addition, we cannot fully exclude that ischemia or other factors related to liver transplantation may have influenced gene expression in our study, as all specimens were collected during liver transplantation. Finally, our study cannot formally clarify whether downregulation of BCKDK is a cause or a consequence of ACLF.

In conclusion, ACLF is characterized by distinct changes in the hepatic transcriptome, which affect key pathways in substrate metabolism and mitochondrial function and are associated with a loss of the functional liver zonation. Downregulation of BCKDK in ACLF might be of particular interest, as it may integrate metabolism of branched-chained amino acids with impaired glucose metabolism and mitochondrial dysfunction. These changes might contribute to the pathogenesis of ACLF-defining organ failures.

Affiliations

[1]*Department of Medicine II, LMU University Hospital, Munich, Germany;* [2]*Department of Visceral Surgery, LMU University Hospital, Munich, Germany;* [3]*Department of Immunodynamics, University of Duisburg-Essen, Duisburg, Germany;* [4]*Institute for Pathology, TUM School of Medicine and Health, Munich, Germany;* [5]*Institute for Cell Biology, University of Duisburg-Essen, Duisburg, Germany;* [6]*Department of Internal Medicine I, Research Center for Immunotherapy (FZI), University Medical Center of the Johannes Gutenberg University Mainz, Mainz, Germany*

Abbreviations

ACLF, acute-on-chronic liver failure; AD, acute decompensation; BCAAs, branched-chain amino acids; BCKDH, branched-chain α-ketoacid dehydrogenase; BCKDK, branched-chain ketoacid dehydrogenase kinase; EASL-CLIF, European Association for the Study of Liver - Chronic Liver Failure; GEO, Gene Expression Omnibus; GSEA, Gene Set Enrichment Analysis; PCA, principal component analysis; RNA-seq, RNA-sequencing; UMAP, uniform manifold approximation and projection.

Financial support

This study was supported by the Deutsche Forschungsgemeinschaft, LA 2806/7-1 and LA 2806/9-1 to CML. DRE received funding from the German Research Foundation: FOR5427 (466687329) SP4; EN984/16-1, 18-1 and 19-1; and TR332 (449437943) A3 and Z1, INST 20876/486-1.

Conflicts of interest

The authors declare no conflicts of interest.

Please refer to the accompanying ICMJE disclosure forms for further details.

Authors' contributions

Study design: MML, HS, BB, CML. Sample collection: MML, HS, BB, CML. Data acquisition: all authors. Interpretation of data: all authors. Statistical analysis: MML, HS, BB, CML. Manuscript writing: MML, HS, BB, CML. Manuscript editing: MM, AB, SJ, DK, MG, DS, DE, CM, JW, KS.

Data availability

RNA sequencing (RNA-seq) data can be accessed at Gene Expression Omnibus (GEO) (accession number: GSE322724). Other data can be obtained from the corresponding author upon resonable request.

Acknowledgements

The authors would like to thank Nadja Lewandowski-Hoppe and Aida Mesinovic for excellent assistance in performing spatial transcriptome analyses.

Supplementary data

Supplementary data to this article can be found online at https://doi.org/10.1016/j.jhepr.2026.102035.

References

Author names in bold designate shared co-first authorship

[1] Moreau R, Jalan R, Gines P, et al. Acute-on-chronic liver failure is a distinct syndrome that develops in patients with acute decompensation of cirrhosis. Gastroenterology 2013;144:1426–1437. 1437.e1421–9.

[2] Trebicka J, Fernandez J, Papp M, et al. The PREDICT study uncovers three clinical courses of acutely decompensated cirrhosis that have distinct pathophysiology. J Hepatol 2020;73:842–854.

[3] Arroyo V, Moreau R, Jalan R. Acute-on-chronic liver failure. N Engl J Med 2020;382:2137–2145.

[4] Zhang IW, Curto A, Lopez-Vicario C, et al. Mitochondrial dysfunction governs immunometabolism in leukocytes of patients with acute-on-chronic liver failure. J Hepatol 2022;76:93–106.

[5] **Arroyo V**, **Angeli P**, **Moreau R**, et al. The systemic inflammation hypothesis: towards a new paradigm of acute decompensation and multiorgan failure in cirrhosis. J Hepatol 2021;74:670–685.

[6] Adebayo D, Morabito V, Andreola F, et al. Mechanism of cell death in acute-on-chronic liver failure: a clinico-pathologic-biomarker study. Liver Int 2015;35:2564–2574.

[7] **Moreau R**, **Claria J**, **Aguilar F**, et al. Blood metabolomics uncovers inflammation-associated mitochondrial dysfunction as a potential mechanism underlying ACLF. J Hepatol 2020;72:688–701.
[8] **Langer MM**, **Eickelmann C**, Kleinbongard P, Lange CM. Impaired mitochondrial complex IV respiration in peripheral blood mononuclear cells discriminates acute-on-chronic liver failure from acute decompensation. J Hepatol 2022;77:874–876.
[9] Claria J, Stauber RE, Coenraad MJ, et al. Systemic inflammation in decompensated cirrhosis: Characterization and role in acute-on-chronic liver failure. Hepatology 2016;64:1249–1264.
[10] Weiss E, de la Pena-Ramirez C, Aguilar F, et al. Sympathetic nervous activation, mitochondrial dysfunction and outcome in acutely decompensated cirrhosis: the metabolomic prognostic models (CLIF-C MET). Gut 2023;72:1581–1591.
[11] Wu H, Yang J, Yang Z, et al. Targeting the BCKDK/BCLAF1/MYC/HK2 axis to alter aerobic glycolysis and overcome Trametinib resistance in lung cancer. Cell Death Differ 2025;32:2210–2224.
[12] Li T, Zhang Z, Kolwicz Jr SC, et al. Defective branched-chain amino acid catabolism disrupts glucose metabolism and sensitizes the heart to ischemia-reperfusion injury. Cell Metab 2017;25:374–385.
[13] **Zhou F**, **Sheng C**, **Ma X**, et al. BCKDH kinase promotes hepatic gluconeogenesis independent of BCKDHA. Cell Death Dis 2024;15:736.
[14] Kim D, Paggi JM, Park C, et al. Graph-based genome alignment and genotyping with HISAT2 and HISAT-genotype. Nat Biotechnol 2019;37:907–915.
[15] Love MI, Huber W, Anders S. Moderated estimation of fold change and dispersion for RNA-seq data with DESeq2. Genome Biol 2014;15:550.
[16] Gu Z, Eils R, Schlesner M. Complex heatmaps reveal patterns and correlations in multidimensional genomic data. Bioinformatics 2016;32:2847–2849.
[17] Blighe K, Rana S, Lewisse M. EnhancedVolcano: publication-ready volcano plots with enhanced colouring and labeling. R package Version 2022;1.16.0.
[18] Pham D, Tan X, Xu J, et al. stLearn:integrating spatial location, tissue morphology and gene expression to find cell types, cell-cell interactions and spatial trajectories within undissociated tissues. Nat Commun 2023;14:7739.
[19] Wolf FA, Angerer P, Theis FJ. SCANPY: large-scale single-cell gene expression data analysis. Genome Biol 2018;19:15.
[20] **Polanski K**, **Young MD**, **Miao Z**, et al. BBKNN: fast batch alignment of single cell transcriptomes. Bioinformatics 2020;36:964–965.
[21] MacParland SA, Liu JC, Ma XZ, et al. Single cell RNA sequencing of human liver reveals distinct intrahepatic macrophage populations. Nat Commun 2018;9:4383.
[22] Harris CR, Millman KJ, van der Walt SJ, et al. Array programming with NumPy. Nature 2020;585:357–362.
[23] McKinney W. Data structures for statistical computing in Python. SciPy 2010. https://proceedings.scipy.org/articles/Majora-92bf1922-00a.
[24] Virtanen P, Gommers R, Oliphant TE, et al. SciPy 1.0: fundamental algorithms for scientific computing in Python. Nat Methods 2020;17:261–272.
[25] Virshup I, Rybakov S, Theis FJ, et al. anndata: Access and store annotated data matrices. J Open Source Softw 2024;9:4371.
[26] Waskom ML. seaborn: statistical data visualization. J Open Source Softw 2021;6:3021.
[27] Hunter JD. Matplotlib: a 2D Graphics Environment. Comput Sci Eng 2007;9:90–95.
[28] **Verbinnen I**, **Boens S**, Ferreira M, et al. Enhanced DNA-repair capacity and resistance to chemically induced carcinogenesis upon deletion of the phosphatase regulator NIPP1. Oncogenesis 2020;9:30.
[29] Krige D, Needham LA, Bawden LJ, et al. CHR-2797: an antiproliferative aminopeptidase inhibitor that leads to amino acid deprivation in human leukemic cells. Cancer Res 2008;68:6669–6679.
[30] Janssen J, Lowenberg B, Manz M, et al. Inferior outcome of addition of the aminopeptidase inhibitor tosedostat to standard intensive treatment for elderly patients with AML and high risk MDS. Cancers (Basel) 2021;13:672.
[31] Oyarzabal A, Bravo-Alonso I, Sanchez-Arago M, et al. Mitochondrial response to the BCKDK-deficiency: some clues to understand the positive dietary response in this form of autism. Biochim Biophys Acta 2016;1862:592–600.
[32] Biswas D, Slade L, Duffley L, et al. Inhibiting BCKDK in triple negative breast cancer suppresses protein translation, impairs mitochondrial function, and potentiates doxorubicin cytotoxicity. Cell Death Discov 2021;7:241.

Keywords: Liver cirrhosis; Spatial transcriptomics; Systemic inflammation; Mitochondrial dysfunction; Branched-chain amino acids; Acute-on-chronic liver failure.
Received 15 April 2026; received in revised form 4 September 2026; accepted 8 September 2026; Available online 10 September 2026

Journal of Hepatology, Volume *8*

Supplemental information

Spatial transcriptomics reveal impaired metabolic liver zonation in acute-on-chronic liver failure

Mona-May Langer, Helena Stadler, Mareike Mannewitz, Alina Bauschen, Severin Jacobi, Dionysios Koliogiannis, Markus Guba, Devon Siemes, Daniel R. Engel, Carolin Mogler, Johannes Wirth, Katja Steiger, Bettina Budeus, and Christian M. Lange

Spatial transcriptomics reveal impaired metabolic liver zonation in acute-on-chronic liver failure

Mona-May Langer, Helena Stadler, Mareike Mannewitz, Alina Bauschen, Severin Jacobi, Dionysios Koliogiannis, Markus Guba, Devon Siemes, Daniel R. Engel, Carolin Mogler, Johannes Wirth, Katja Steiger, Bettina Budeus, Christian M. Lange

Table of contents

Fig. S1………………………………………………………………………………………2

Fig. S2………………………………………………………………………………………3

Fig. S3………………………………………………………………………………………4

Fig. S4………………………………………………………………………………………5

Table S1……………………………………………………..…………separate excel file

Table S2……………………………………………………………………………………6

Table S3……………………………………………………………………………………7

Table S4……………………………………………………………………………………8

Table S5……………………………………………………………………………………8

Fig. S1: Heatmap showing unsupervised clustering of the top differentially regulated genes (DRGs) across stages of cirrhosis (padj < 0.03 & abs(log2FC) > 0.65).

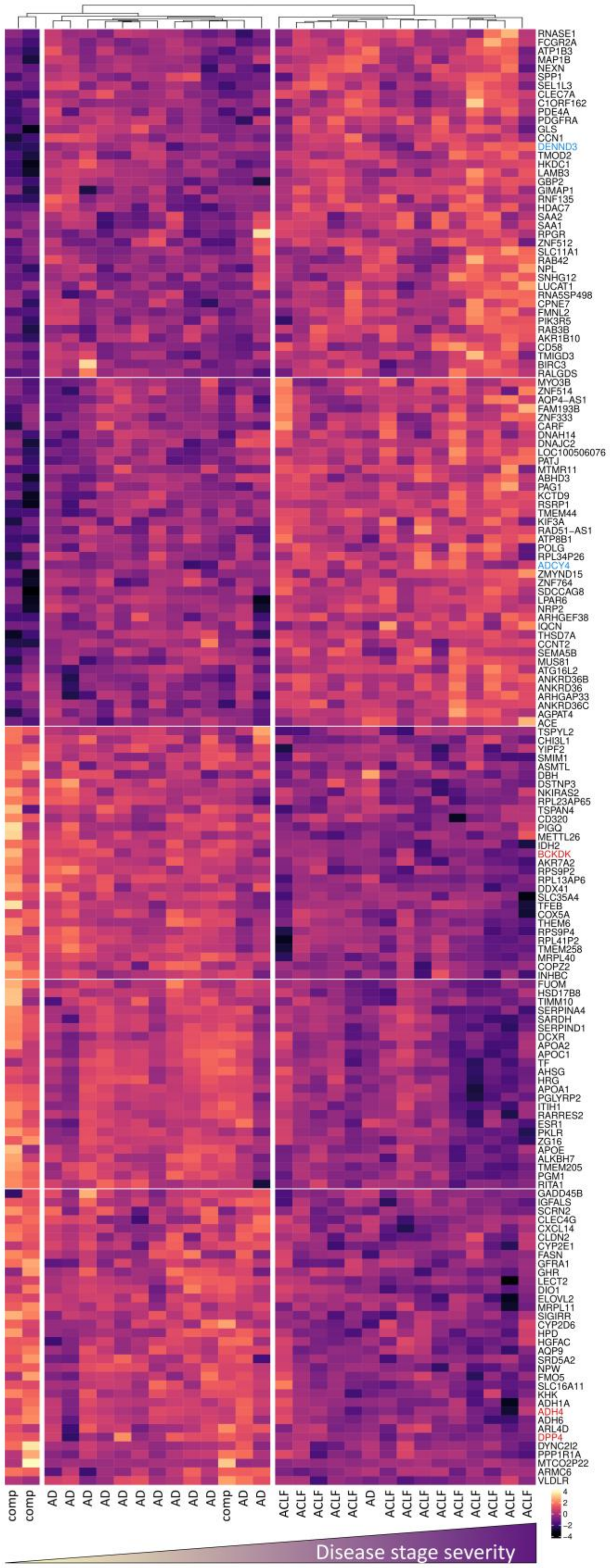

Fig. S2: Correlation of hepatic expression of selected genes with markers of systemic inflammation.

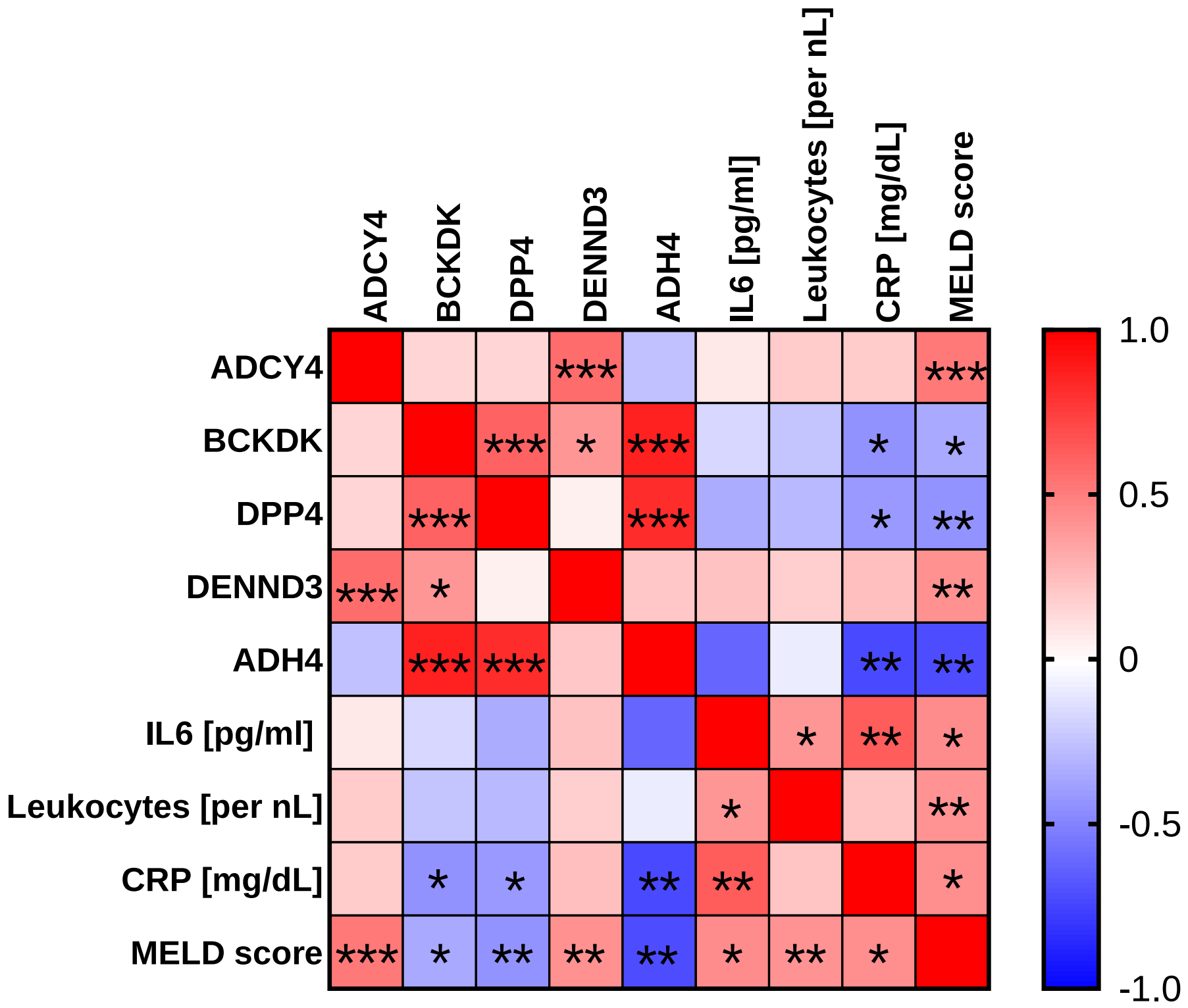

Correlations of hepatic gene expression, assessed by quantitative PCR (see Figure 1) with markers of systemic inflammation. *P ≤ 0.05, **P ≤ 0.01, ***P ≤ 0.001.

Fig. S3: Association between hepatic expression of selected genes according to the presence of infections as precipitants of ACLF.

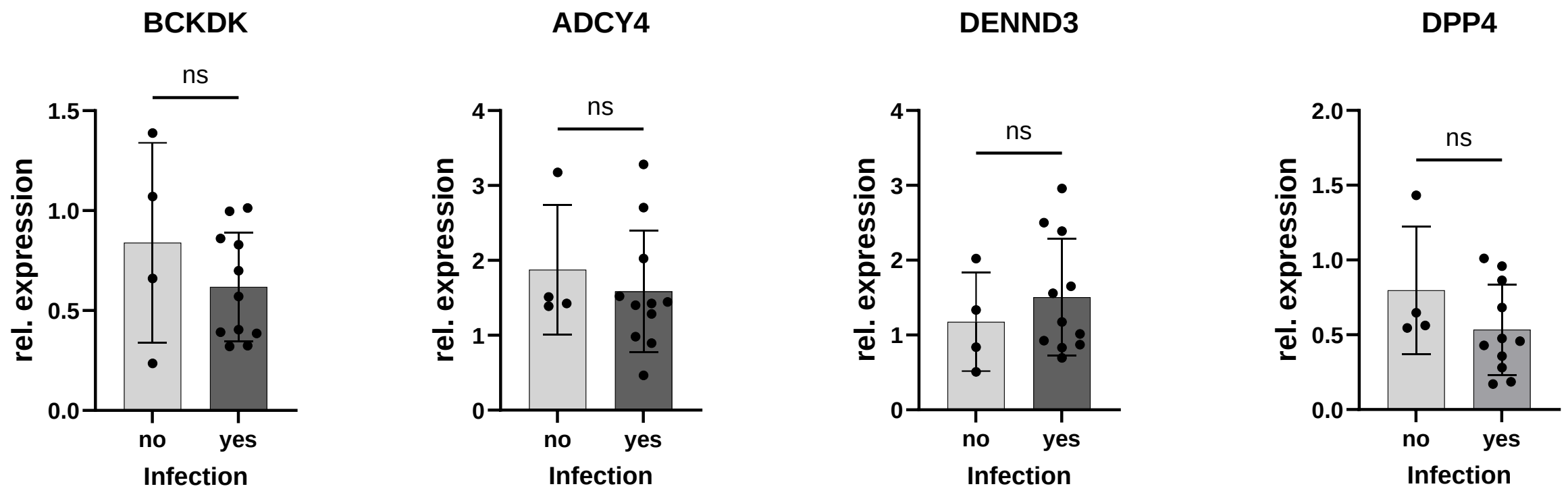

Data are shown for patients with ACLF with versus without infections.

Fig. S4: Association between hepatic expression of selected genes according to the type of organ failures.

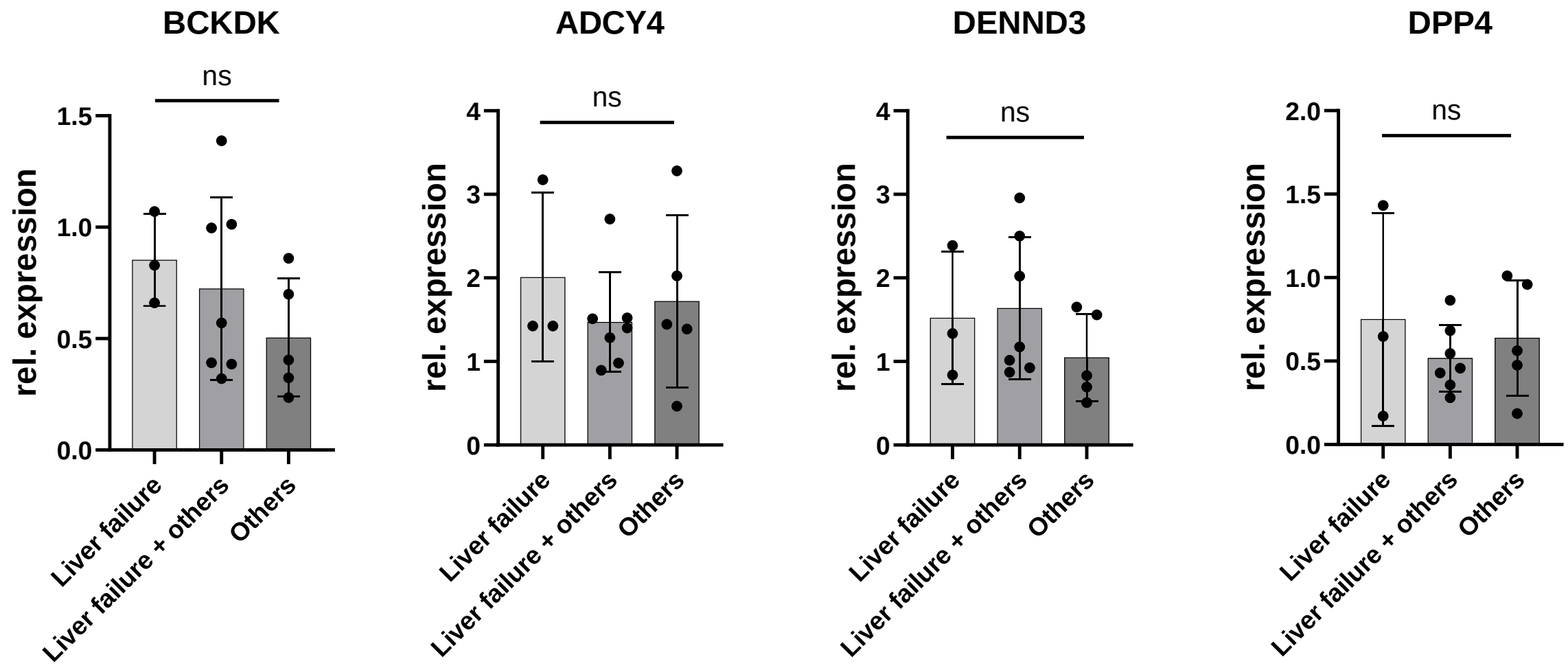

Data are shown for patients with ACLF with versus without indicated organ failures.

Table S1. List of genes included in Xenium Panel: See attached Excel sheet.

Table S2. List of genes (according to (21)) used for cell type annotation of spatial transcriptome analysis.

Cell Type	Marker Genes
Hepatocytes_drugmetabolism	ADH4, CYP3A4, RPP25L, HAMP, BCHE, G6PC
Hepatocytes_gluconeogenesis	ADH4, CYP3A4, SCD, HMGCS1
Hepatocytes_immuneactivation	ADH4, CYP3A4, SEC16B, CYP2A7
LSECs_Zone1	RAMP2, CLEC14A, VWF, C7, SRPX, CAV1, SOX18
LSECs_Zone2-3	FCN2, CRHBP, GNG11, DNASE1L3, ADGRL4, EGFL7, LYVE1
non-LSEC_endothelial_cells	PECAM1, INMT, DNASE1L3, PTGDS, C7, VWF, SRPX
Cholangiocytes	EPCAM, TFF2, FXYD2, KRT7, CXCL6
HSC	ACTA2, PDGFRB, LTBP2, HGF
Kupffer_cells	CD163, MARCO, MRC1, FCN1
B_cells	CD19, CD27, MS4A1, TCL1A, BANK1, IRF8, CD79A
Dendritic_cells	CD14, CD19, CD80, CD83, CD86, IRF8, LILRA4
Macrophages	IL1B, IL6, CD80, CD86, CD4, CD83
T_cells	CD2, CD3D, TRAC, GZMK, CCL5, GZMA, CD3E, CD69, IL7R, DUSP2
unconventional_T_cells	CD3D, KLRB1, FCGR3A, NKG7, GNLY, PTGDS, GZMB, PRF1, KLRD1, GZMA, CCL5
NK-like_cells	GZMK, KLRB1, KLRC1, KLRD1, NKG7, CD69, DUSP2, CD247
NK_cells	KLRC1, GNLY, PRF1
Neutrophils	CEACAM8, FUT4, ITGAM, FCGR3A

Table S3. Primers used for quantitative PCR.

Target	Forward primer (5'-3')	Reverse primer (5'-3')
BCKDK	GCCACAATGGAGAGTCACCTAG	CTTTGTGAGCGATTCCTCCACC
ADCY4	GAGCCTACCTATCTGGTCATCG	GACTCCAGGTAACGGGTCATCA
DENND3	TCGTGAAGGACGCCAGCATCAT	TCCGTCTCCTTCCAGCAGTTGT
ADH4	CCTTGACTGTGCAGGTGGATCT	GTCAATCCTTTGCTACCAGCAGC
DPP4	AAAGGCACCTGGGAAGTCATCG	CAGCTCACAACTGAGGCATGTC

Table S4. Types of organ failure in patients with ACLF (N = 15)

Organ failure	N (%)
Liver failure	10 (66.7)
Kidney failure	9 (60.0)
Cerebral failure	0 (0.0)
Coagulation failure	2 (13.3)
Circulation failure	8 (53.3)
Respiratory failure	4 (26.7)

Data are presented as N (%) of the total ACLF cohort (N = 15).

Table S5. Types of infections in patients according to disease stage

Type of infection	Comp (n=9)	AD (n=15)	ACLF (n=15)	Total (N=39)
Any infection	0 (0.0)	1 (6.7)	11 (73.3)	12 (30.8)
Type of infection				
Spontaneous bacterial peritonitis (SBP)	0 (0.0)	0 (0.0)	3 (20.0)	3 (7.7)
Pneumonia	0 (0.0)	0 (0.0)	2 (13.3)	2 (5.1)
Urinary tract infection	0 (0.0)	0 (0.0)	2 (13.3)	2 (5.1)
Skin and soft tissue infection	0 (0.0)	0 (0.0)	2 (13.3)	2 (5.1)
Intra-abdominal abscess	0 (0.0)	0 (0.0)	1 (6.7)	1 (2.6)
Bacteremia without identified primary focus	0 (0.0)	0 (0.0)	1 (6.7)	1 (2.6)
Suspected infection without confirmed focus	0 (0.0)	1 (6.7)	1 (6.7)	2 (5.1)

Data are presented as N (%). One patient with ACLF had infections at two anatomically distinct sites and is counted in both corresponding categories
