# Supplementary material for: Spatial transcriptomics reveal impaired metabolic liver zonation in acute-on-chronic liver failure
Source: JHEP Rep. 2026 Sep 10;8(12):102035. doi: 10.1016/j.jhepr.2026.102035 (PMC13756263; doi:10.1016/j.jhepr.2026.102035)
Supplement: Supplementary file 4 [file mmc2.docx]

**JHEP Reports**

**CTAT methods**

Tables for a “Complete, Transparent, Accurate and Timely account” (CTAT) are now mandatory for all revised submissions. The aim is to enhance the reproducibility of methods.

- Only include the parts relevant to your study
- Refer to the CTAT in the main text as ‘Supplementary CTAT Table’
- Do not add subheadings
- Add as many rows as needed to include all information
- Only include one item per row

**If the CTAT form is not relevant to your study, please outline the reasons why:**

-

1.1 **Antibodies**

| **Name** | **Citation** | **Supplier** | **Cat no.** | **Clone no.** |
| --- | --- | --- | --- | --- |
| Phospho-BCKDH-E1 alpha (Ser293) (E2V6B) Rabbit Monoclonal Antibody | Cell Signaling Technology Cat# 40368, RRID:AB_2799176 | Cell Signaling Technology (Danvers, USA) | 40368S | E2V6B |
| BCKDH-E1 alpha (E4T3D) Rabbit Monoclonal Antibody | Cell Signaling Technology Cat# 90198, RRID:AB_2800155 | Cell Signaling Technology (Danvers, USA) | 90198S | E4T3D |
| BCKDK (E5B6J) Rabbit Monoclonal Antibody | Cell Signaling Technology Cat# 82318 | Cell Signaling Technology (Danvers, USA) | 82318S | E5B6J |
| Anti-β-Actin (ACTB) Antibody  mouse monoclonal | Sigma-Aldrich Cat# A1978, RRID:AB_476692 | Merck KGaA (Darmstadt, Germany) | A1978-200UL | AC-15 |
| Mouse anti-rabbit IgG-HRP | (Santa Cruz Biotechnology Cat# sc-2357, RRID:AB_628497) | Santa Cruz Biotechnology (Dallas, USA) | sc-2357 | Monoclonal |
| Goat anti-mouse IgG-AP | (Santa Cruz Biotechnology Cat# sc-2047, RRID:AB_631732) | Santa Cruz Biotechnology (Dallas, USA) | sc-2047 | Polyclonal |

1.2 **Cell lines**

| **Name** | **Citation** | **Supplier** | **Cat no.** | **Passage no.** | **Authentication test method** |
| --- | --- | --- | --- | --- | --- |
| Human Hepatocytes, Cryopreserved, Plateable | - | Lonza (Basel, Switzerland) | HUCPG,  single donor, Batch No. 24TL071867 | Primary cells (P0) | Supplier Certificate of Analysis (Thaw viability, patient characteristics,CYP-activity |

1.3 **Organisms**

| **Name** | **Citation** | **Supplier** | **Strain** | **Sex** | **Age** | **Overall n number** |
| --- | --- | --- | --- | --- | --- | --- |
| **-** | **-** | **-** | **-** | **-** | **-** | **-** |

1.4 **Sequence based reagents**

| **Name** | **Sequence** | **Supplier** |
| --- | --- | --- |
| BCKDH kinase (BCKDK), Human, forward | 5'-GCCACAATGGAGAGTCACCTAG-3' | Sequence: OriGene Technologies; Synthesized by: Eurofins Genomics |
| BCKDH kinase (BCKDK), Human, reverse | 5'-CTTTGTGAGCGATTCCTCCACC-3' | Sequence: OriGene Technologies; Synthesized by: Eurofins Genomics |
| ADCY4, Human, forward | 5'-GAGCCTACCTATCTGGTCATCG-3' | Sequence: OriGene Technologies; Synthesized by: Eurofins Genomics |
| ADCY4, Human, reverse | 5'-GACTCCAGGTAACGGGTCATCA-3' | Sequence: OriGene Technologies; Synthesized by: Eurofins Genomics |
| DENND3, Human, forward | 5'-TCGTGAAGGACGCCAGCATCAT-3' | Sequence: OriGene Technologies; Synthesized by: Eurofins Genomics |
| DENND3, Human, reverse | 5'-TCCGTCTCCTTCCAGCAGTTGT-3' | Sequence: OriGene Technologies; Synthesized by: Eurofins Genomics |
| CD26 (DPP4), Human, forward | 5'-AAAGGCACCTGGGAAGTCATCG-3' | Sequence: OriGene Technologies; Synthesized by: Eurofins Genomics |
| CD26 (DPP4), Human, reverse | 5'-CAGCTCACAACTGAGGCATGTC-3' | Sequence: OriGene Technologies; Synthesized by: Eurofins Genomics |
| ADH4, Human, forward | 5'-CCTTGACTGTGCAGGTGGATCT-3' | Sequence: OriGene Technologies; Synthesized by: Eurofins Genomics |
| ADH4, Human, reverse | 5'-GTCAATCCTTTGCTACCAGCAGC-3' | Sequence: OriGene Technologies; Synthesized by: Eurofins Genomics |

1.5 **Biological samples**

| **Description** | **Source** | **Identifier** |
| --- | --- | --- |
| Human liver tissue from explanted livers of patients with liver cirrhosis (compensated, decompensated, acute-on-chronic liver failure, collected during liver transplantation | Klinikum der Ludwig-Maximilians-Universität München, Department of General, Visceral and Transplant Surgery (surgical procurement), and Department of Medicine II (study coordination), Munich, Germany | LMU 22-0392 |

1.6 **Deposited data**

| **Name of repository** | **Identifier** | **Link** |
| --- | --- | --- |
| Gene Expression Omnibus (GEO) | GSE322724 | https://www.ncbi.nlm.nih.gov/geo/query/  acc.cgi?acc=GSE322724 |

1.7 **Software**

| **Software name** | **Manufacturer** | **Version** |
| --- | --- | --- |
| GraphPad Prism | GraphPad Software (Boston, USA) | 9.0.2 |
| Xenium Analyzer instrument software | 10x Genomics (Pleasanton, USA) | 4.0.1.4 |
| Xenium Explorer | 10x Genomics (Pleasanton, USA) | 4.0.1.0 |
| Python | Python Software Foundation (Beaverton, USA) | 3.9.21 |
| R | R Core Team, R Foundation for Statistical Computing (Vienna, Austria) | 4.5.1 |
| Fiji (ImageJ) | Schindelin et al., Nat Methods 2012 (open-source) | 1.53e |

1.8 **Other (*e.g*. drugs, proteins, vectors etc.)**

| **Name** | **Supplier** | **Cat no.** |
| --- | --- | --- |
| RNAlater™ Stabilization Solution | Invitrogen (Thermo Fisher) (Waltham, USA) | AM7020 |
| RNeasy Mini Kit | Qiagen (Hilden, Germany) | 74106 |
| QuantSeq 3' mRNA-Seq Library Prep Kit FWD | Lexogen (Vienna, Austria) | N/A |
| PrimeScript™ RT Reagent Kit | Takara Bio (Kusatsu, Japan) | RR037A |
| TB Green® Premix Ex Taq™ | Takara Bio (Kusatsu, Japan) | RR42WR |
| RIPA Buffer (10X) | Cell Signaling Technology (Danvers, USA) | 9806S |
| cOmplete™, Mini, EDTA-free Protease Inhibitor Cocktail | Roche Diagnostics (Mannheim, Germany) | 11836170001 |
| PhosSTOP™ Phosphatase Inhibitor Cocktail Tablets | Roche Diagnostics (Mannheim, Germany) | 4906837001 |
| Bio-Rad Protein Assay Kit I | Bio-Rad Laboratories (Hercules, USA) | 5000001 |
| Immobilon-P Membran, PVDF, 0,45 µm | Millipore (Billerica, USA) | IPVH00010 |
| Immobilon Forte Western HRP substrate | Merck KGaA (Darmstadt, Germany) | WBLUF0100 |
| ReBlot Plus Strong Antibody Stripping Solution | Merck KGaA (Darmstadt, Germany) | 2504 |
| Collagen I Cell Culture Surface Coating Kit | Innoprot(Derio, Spain) | P8188 |
| Human Cryopreserved Hepatocyte Thawing Medium | Lonza (Basel, Switzerland) | MCHT50 |
| Hepatocyte Plating Medium | Lonza (Basel, Switzerland) | MP100 |
| HCM^TM^ Hepatocyte Culture Medium BulletKit® | Lonza (Basel, Switzerland) | CC-3198 |
| BT2 (BCKDK Inhibitor), 50mg | MedChemExpress (Monmouth Junction, USA) | HY-114855 |
| pCPT-cAMP 10MG | Merck KGaA (Darmstadt, Germany) | C3912-10MG |
| Glucose Colorimetric Detection kit | Invitrogen (Thermo Fisher) (Waltham, USA) | EIAGLUC |

1.9 **Please provide the details of the corresponding methods author for the manuscript:**

Prof. Dr. Christian M. Lange,

Department of Internal Medicine I, University Medical Center of the Johannes Gutenberg University Mainz, Langenbeckstr. 1, D-55131 Mainz, Germany.

Phone: 0049 6131 177276. Fax: 0049 6131 175595.

E-Mail: Christian.Lange@unimedizin-mainz.de

**2.0	Please confirm for randomised controlled trials all versions of the clinical protocol are included in the submission. These will be published online as supplementary information.**

**-**
